# Penicillinase-resistant antibiotics induce non-immune-mediated cholestasis through HSP27 activation associated with PKC/P38 and PI3K/AKT signaling pathways

**DOI:** 10.1038/s41598-017-01171-y

**Published:** 2017-05-12

**Authors:** Audrey Burban, Ahmad Sharanek, Romain Hüe, Marion Gay, Sylvain Routier, André Guillouzo, Christiane Guguen-Guillouzo

**Affiliations:** 1 INSERM U991, Liver Metabolisms and Cancer, Rennes, France; 2 Rennes 1 University, Rennes, France; 3 ICOA, University of Orleans, UMR CNRS 7311, Orléans, France

## Abstract

The penicillinase-resistant antibiotics (PRAs), especially the highly prescribed flucloxacillin, caused frequent liver injury via mechanisms that remain largely non-elucidated. We first showed that flucloxacillin, independently of cytotoxicity, could exhibit cholestatic effects in human hepatocytes in the absence of an immune reaction, that were typified by dilatation of bile canaliculi associated with impairment of the Rho-kinase signaling pathway and reduced bile acid efflux. Then, we analyzed the sequential molecular events involved in flucloxacillin-induced cholestasis. A crucial role of HSP27 by inhibiting Rho-kinase activity was demonstrated using siRNA and the specific inhibitor KRIBB3. HSP27 activation was dependent on the PKC/P38 pathway, and led downstream to activation of the PI3K/AKT pathway. Other PRAs induced similar cholestatic effects while non PRAs were ineffective. Our results demonstrate that PRAs can induce cholestatic features in human hepatocytes through HSP27 activation associated with PKC/P38 and PI3K/AKT signaling pathways and consequently support the conclusion that in clinic they can cause a non-immune-mediated cholestasis that is not restricted to patients possessing certain genetic determinants.

## Introduction

Different classes of marketed drugs and herbals have been reported to cause drug-induced liver injury (DILI) in humans, accounting for more than 50% of cases of acute liver failure in the United States^1^. Antibiotics represent the most common causes of DILI^2, 3^ and have been associated with high rate of morbidity as well as many cases of liver transplantation and death resulting from acute liver failure^4–6^. Antibiotic-induced hepatotoxicity is mostly idiosyncratic and can occur through an immunological reaction or in response to generation of reactive metabolites and/or formation of protein adducts. Its frequency depends on the antibiotic: while DILI is rarely observed with certain penicillin derivatives such as penicillin G or V, ampicillin and amoxicillin^7^, cholestatic hepatitis is frequently induced by the semi-synthetic penicillinase-resistant antibiotics (PRAs) such as cloxacillin, nafcillin, and most notably flucloxacillin (FLX)^8^.

FLX is a highly prescribed semi-synthetic β-lactam PRA for staphylococcal infections. It is estimated to cause cholestasis liver injury in ~8/100,000 patients^9^, making it a significant medical problem^10^. FLX is the most common reason of idiosyncratic liver injury in Sweden, with 16% of all DILI cases and the second most common cause of drug-induced cholestasis in the United Kingdom^11, 12^. Mechanisms underlying FLX-induced hepatocellular injury and cholestasis remain non-elucidated. It is assumed that occurrence of liver injury in patients under FLX treatment is caused by immune-mediated response and favored by genetic determinants^9, 13^. However, there is no evidence of the implication of such reactions in the development of cholestasis due to FLX. A genome-wide association study has revealed the human leukocyte antigen (HLA)-B*57:01 genotype as a major determinant of FLX-induced liver injury^14^. Nevertheless, symptoms consistent with hypersensitivity have not been observed in a substantial proportion of patients and this might suggest that non-immune mechanisms may also be operative in the hepatocyte^13^; however, this has never been investigated. Thus, the potential of PRAs to induce directly cholestasis in a metabolically competent hepatic cell model that lacks the immune factors would be appreciable. *In vivo* studies have demonstrated that treatment with FLX resulted in the formation of hepatic protein adducts^15^. Many hepatotoxicants have been demonstrated to produce reactive metabolites that bind covalently to liver proteins^16, 17^. Interestingly, toxicant-adducted proteins could stimulate specific members of the heat shock protein (HSP) family that are thought to chaperone these non-native proteins leading to protection against cell death^18^.

HSP27, a member of the HSP family, is characterized by its dynamic phosphorylation leading to heterogeneous oligomerization under different conditions such as oxidative stress, heat shock as well as chemical stress^19^. HSP27 is important in many cell functions. It is known to be involved in cell movement by regulating the polymerization of actin^20^. Phosphorylation of HSP27 is essential in actin cytoskeleton organization and actin-dependent events in response to growth factors and stress and for its interaction with Rho-kinase (ROCK)^21–23^. Activated ROCK leads to myosin phosphatase target subunit 1 (MYPT1) phosphorylation and myosin light chain phosphatase inhibition resulting in myosin activation and acto-myosin contraction. Recent studies from our laboratory showed that alterations of bile canaliculi (BC) dynamics that are associated with bile secretion failure are major features of drug-induced cholestasis^24^. Disruption of Rho/Myosin light chain kinases (ROCK/MLCK) signaling pathway represents crucial mechanisms that underlie cytoskeleton rearrangement and BC deformations accompanying cholestatic insults^24^. Thus, it is worthwhile to explore whether HSP27 is involved in PRA-induced cholestasis.

Activity of HSP27 protein is regulated by its phosphorylation. Previous studies showed that HSP27 phosphorylation could be induced by P38 activation. In addition, HSP27 has been found to direct chaperoning interaction with protein kinase B (AKT)^19^. HSP27 and AKT can form a complex with p38 mitogen-activated protein kinase (MAPK)^25^. Activation of p38 MAPK results in the downstream phosphorylation of HSP27 that can bind to AKT and acts as a scaffold protein to permit phosphorylation of AKT by phosphoinositide 3-kinase (PI3K) in order to protect against cell death by blocking apoptosis. Generally, PI3K/AKT is a protective mechanism recruited by the cells to adapt against stress^26, 27^. Therefore, it might be questioned as to whether this adaptive stress response could contribute to liver cholestasis induced by PRAs.

In the current study, we first investigated the direct cholestatic potential of FLX in the hepatocytes using the metabolically competent human HepaRG and primary human hepatocytes (PHH). Then, we focused on the cascade of events that underlie FLX-induced cholestasis. We evidenced a correlation between HSP27 phosphorylation, ROCK inhibition and induction of cholestatic features associated with PKC/P38 and PI3K/AKT activation. These observations were extended to other frequently used antibiotics of the same family.

## Results

### Cytotoxicity effects of FLX

**Figure 1:**
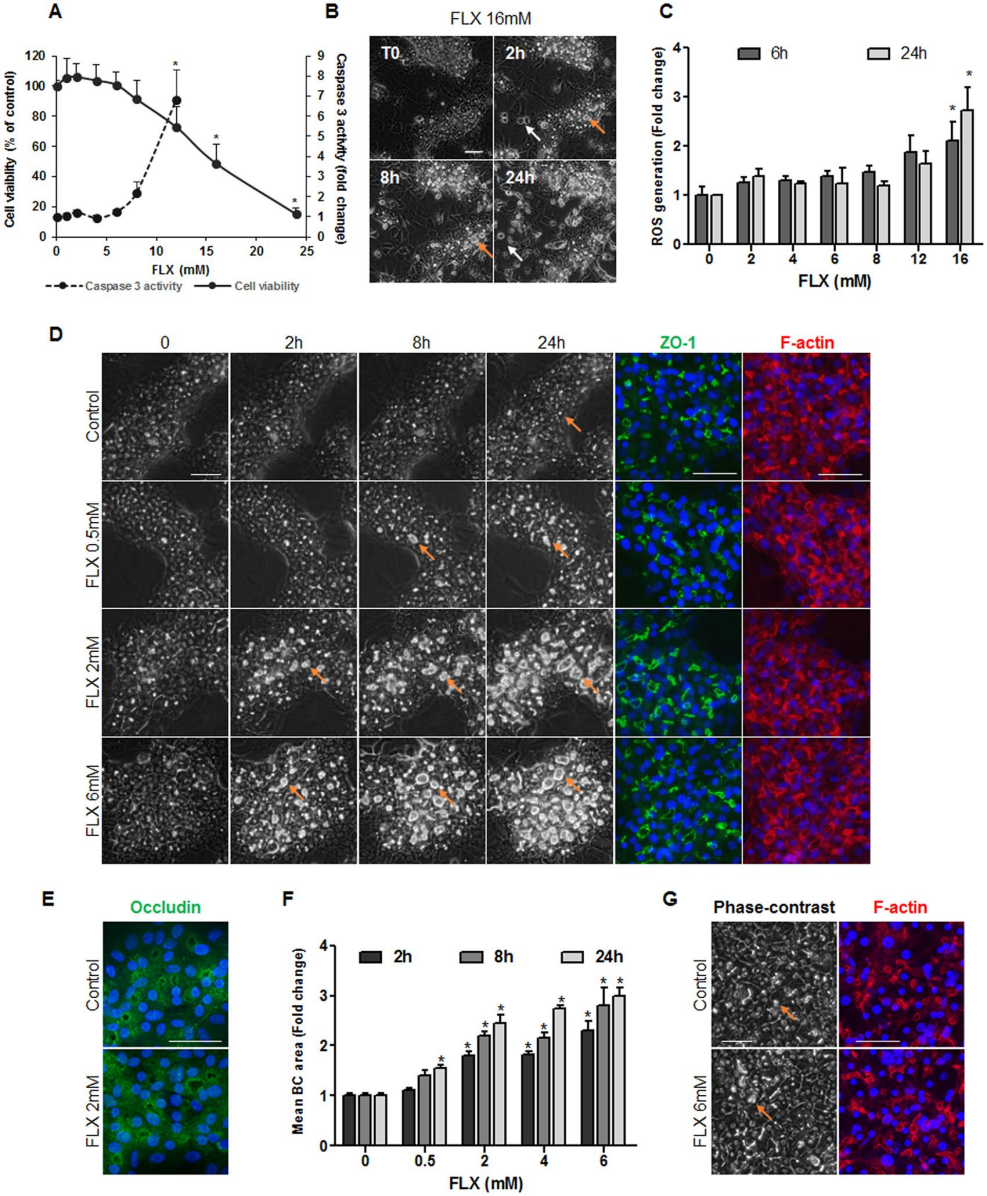
Cytotoxicity and alteration of BC structures by FLX in human HepaRG cells and primary hepatocytes. (**A**) HepaRG cells were incubated with different concentrations of FLX (0–24 mM) for 24 h. Cytotoxicity was measured using the MTT colorimetric and caspase-3 activity assays. (**B**) Representative phase-contrast images of 16 mM FLX-treated HepaRG cells showing higher sensitivity of primitive biliary-like cells (white arrows) than hepatocytes. (**C**) HepaRG cells were treated with different concentrations of FLX for 6 or 24 h. ROS generation was detected by the DCFDA specific substrate. (**D**) HepaRG cells were incubated with different non cytotoxic concentrations of FLX (0–6 mM) at different time points. Immunolabelling of the junctional ZO-1 protein (green) in HepaRG cells treated for 4 h with FLX compared with control cells. F-actin was localized using rhodamine-phalloidin fluoroprobe (red). (**E**) Immunolabelling of the junctional protein occludin (green) in HepaRG cells treated with 2 mM FLX compared with control cells. (**F**) Quantification of BC area after 2, 8 and 24 h using ImageJ 1.48 software as described in the Materials and Methods. Data were expressed as the fold change in the BC mean area relative to the mean area of untreated cells arbitrarily set at a value of 1. They represented the means ± SEM of 3 independent experiments. *p < 0.05 compared with that of untreated cells. (**G**) Phase-contrast images and F-actin localization (red) in PHH treated with 6 mM FLX for 2 h. Nuclei stained in blue (Hoechst dye). Phase-contrast images were captured using time lapse microscopy. Orange arrows indicate BC. The fluorescent images were obtained with a Cellomics ArrayScan VTI HCS Reader (bar = 50 µm).

Cytotoxicity of FLX was evaluated in HepaRG cells using the MTT test. FLX did not cause any significant cytotoxicity after 24 h at concentrations up to 12 mM. However, at 16 mM it caused a 40% decrease in cell viability. Caspase-3 activity was also analyzed; FLX did not increase caspase-3 activity at concentrations up to 6 mM (Fig. 1A). Accordingly, phase-contrast microscopic images showed no cytotoxicity at concentrations lower than 12 mM. By contrast, at 16 mM or higher concentrations cell detachment was observed. Noteworthy, the primitive biliary-like population of HepaRG cells was more sensitive than the HepaRG hepatocyte population (Fig. 1B).

Protein levels of 4 inflammatory stress markers, i.e. interleukin-1β (Il-1 β), interleukin-6 (IL-6), interleukin-8 (IL-8) and C-reactive protein (CRP) were measured after 24 h treatment with 0–8 mM FLX. IL-6 and IL-1 were not detected (data not shown) whereas IL-8 and CRP levels were dose-dependently reduced in the supernatant of FLX-treated cell cultures, indicating an absence of inflammatory response to the antibiotic (Supplementary Fig. 1).

Since deregulation of the cellular redox status is a potent mechanism in DILI, generation of oxidative stress was analyzed after 6 and 24 h of exposure to FLX. Reactive oxygen species (ROS) production was observed with FLX at concentrations higher than 12 mM (Fig. 1C). Transcript levels of two ROS markers (HO-1 and MnSOD) were measured after 6 h of exposure to FLX. Whereas no significant modulation of MnSOD was observed, HO-1 was found to be significantly up-regulated reaching 3-fold with 8 mM FLX. Moreover, four ER stress-related genes (ATF4, ATF6, GRP78 and CHOP) were also analyzed and found to be up-regulated, except ATF6, after 6 h treatment with 8 mM FLX (Supplementary Table 1). Based on cytotoxicity and ROS generation data, FLX was used at 0–6 mM for further studies.

### FLX induces direct cholestatic effects in hepatocytes

#### FLX induces BC deformation in HepaRG cells and PHH

Using time-lapse microscopy, BC morphology was examined during 24 h after FLX addition in both HepaRG cells and PHH. Phase-contrast imaging showed that exposure to FLX resulted in a progressive dilatation of BC in a dose-dependent manner (Fig. 1D and E). At 2 to 6 mM, FLX induced ≈ 2-fold increase in BC dilatation as early as 2 h to reach progressively ≈ 3-fold after 24 h with 6 mM, that represented a mean area of 222 μm^2^ in 6 mM FLX-treated cells compared to 74 μm^2^ in untreated cells. At 0.5 mM FLX caused BC dilatation only after 8 h (Fig. 1D and E). Similar dilatations of BC were observed in FLX-treated PHH (Fig. 1F). BC deformations were confirmed by rhodamine-phalloidin staining of pericanalicular F-actin. The junctional proteins, zona occludens-1 (ZO-1) and occludin, were immunolocalized and revealed punctuated distribution along the canalicular membranes that were comparable between untreated and FLX-treated cells, suggesting that tight junctions were not altered by FLX treatment (Fig. 1D and E).

#### Bile flow alteration in FLX-treated HepaRG cells and PHH

**Figure 2:**
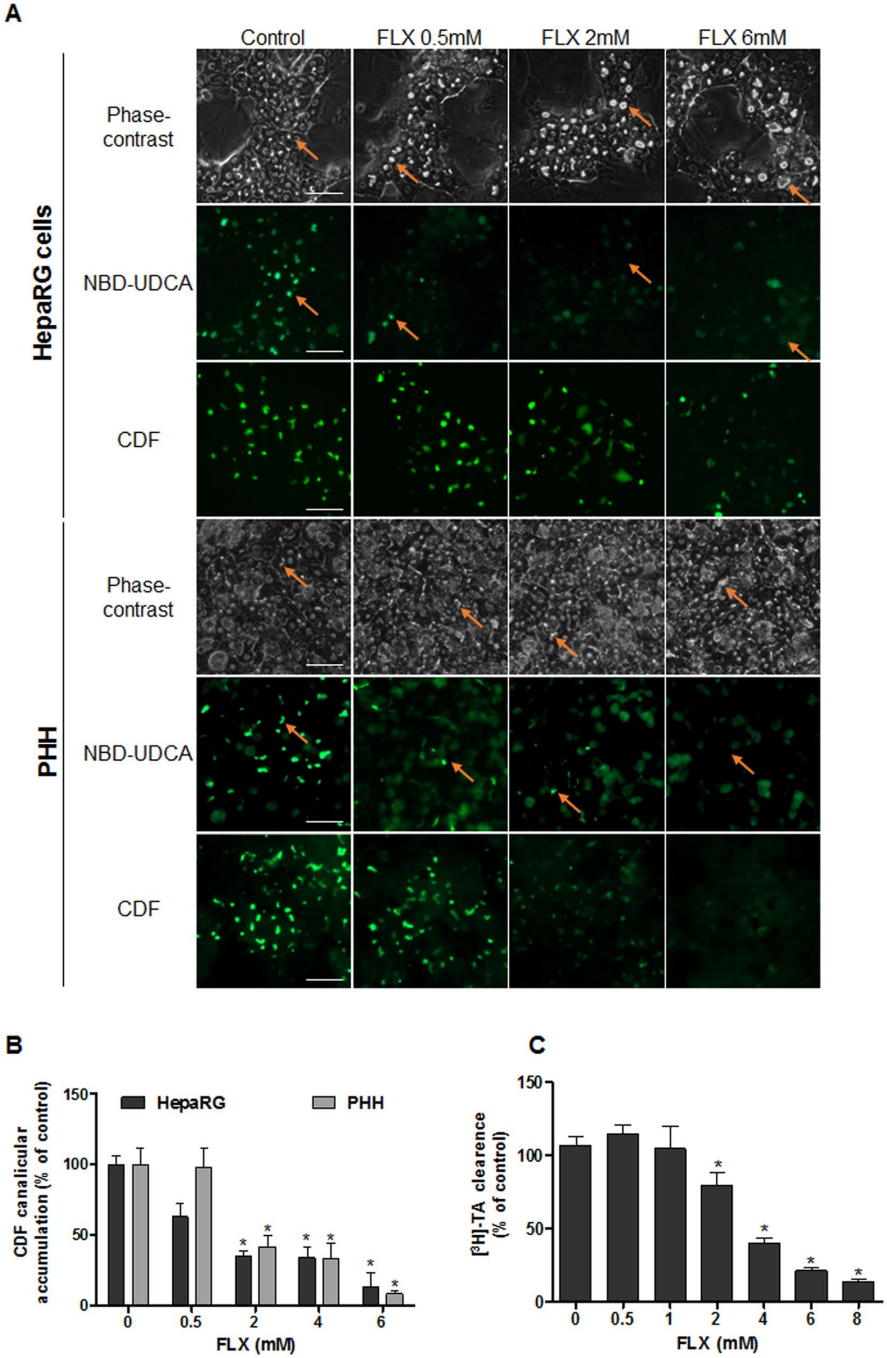
Effects of FLX on labelled bile acids and CDF clearance in HepaRG cells and PHH. (**A**) NBD-UDCA and CDF efflux in HepaRG hepatocytes and PHH treated for 2 h with different concentrations of FLX (0–6 mM). Orange arrows indicate fluorescence in BC (bar = 50 μm). (**B**) Quantification of CDF accumulation in BC of HepaRG hepatocytes after FLX treatment using ImageJ 1.48 software as described in the Materials and Methods. (**C**) [^3^H]-TA clearance in HepaRG cells treated with different concentrations of FLX (0–8 mM) for 2 h. Data were expressed relative to those of untreated cells arbitrarily set at 100%. They represent the means ± SEM of 3 independent experiments. *p < 0.05 compared with that of untreated cells.

As BC deformations could be associated with failure in bile flow, we analyzed if FLX disrupted efflux activity using two fluorescent probes, CDF and NBD-UDCA, substrates of MRP2 and BSEP respectively. After 2 h both fluorescent substrates were visualized in the BC lumen of untreated cells, whereas no well-defined canalicular labeling was observed in dilatated BC of FLX-treated cells at concentrations greater than 2 mM. A dose-dependent decrease in canalicular CDF accumulation, reaching 35% and 42% in HepaRG cells and PHH respectively, was observed with 2 mM FLX (Fig. 2A and B) after 2 h treatment. Effect of FLX on clearance of [^3^H]-TA, mainly transported by BSEP, was also assessed. While no change was observed in [^3^H]-TA clearance after 2 h with 1 mM FLX treatment, a 35% and 87% significant decrease in [^3^H]-TA efflux was measured in HepaRG cells with 2 and 8 mM FLX respectively (Fig. 2C).

A set of genes encoding hepatobiliary transporters was analyzed by RT-qPCR after 6 and 24 h treatment with FLX at 0.5–8 mM. After 6 h only MDR1 gene was up-regulated. Later on, after 24 h BSEP, MDR3 and NTCP were found to be strongly inhibited in a dose-dependent manner, whereas MRP2, MRP4 and MDR1 were up-regulated. By contrast, MRP3 expression was not significantly modulated (Supplementary Table 1). These delayed alterations in gene expression could represent secondary feedback regulation in response to the cholestatic insult.

#### Deregulation of the ROCK pathway

**Figure 3:**
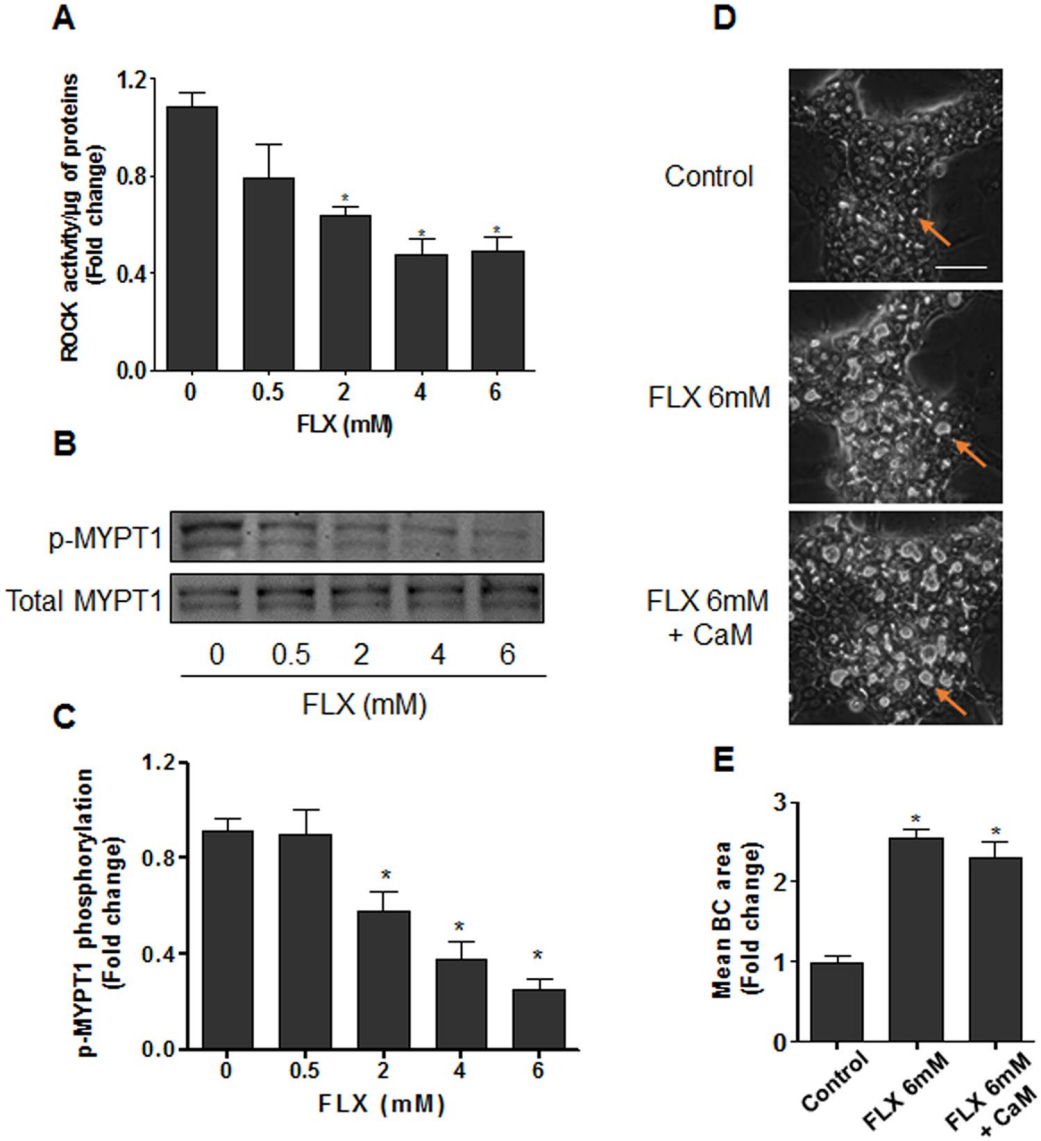
Alteration of ROCK activity and MYPT1 phosphorylation by FLX. (**A**) HepaRG cells were treated with FLX (0–6 mM) for 4 h, then ROCK activity was assessed using a ROCK activity assay Kit (Millipore, catalogue CSA001). (**B**) Representative western blots of p-MYPT1/total MYPT1 after 4 h FLX (0–6 mM) treatment. The displayed blots were cropped and the original full-length gels are included in the supplementary information. (**C**) Quantification of MYPT1 phosphorylation using ImageJ 1.48 software. (**D**) Representative phase-contrast images of HepaRG cells treated with 6 mM FLX alone or combined with 5 µM CaM, a MLCK activator. Orange arrows indicate BC (bar = 50 µm). (**E**) Quantification of BC area after 4 h using ImageJ 1.48 software. Data were expressed relative to those of the untreated cells arbitrary set at a value of 1. They represent the means ± SEM of 3 independent experiments. *p < 0.05 compared with that of controls.

We further analyzed whether FLX-induced BC deformations were associated with alteration of ROCK, a target of cholestatic drugs^24^. Treatment with 0.5–6 mM FLX decreased ROCK activity in a dose-dependent manner after 4 h treatment (Fig. 3A). Modulation of ROCK activity by FLX was further confirmed by analyzing the phosphorylation state of the regulatory/myosin binding subunit (MYPT1), a downstream substrate of ROCK. Indeed, a dose-dependent decrease in MYPT1 phosphorylation associated with BC dilatation was observed while total MYPT1 content remained unchanged (Fig. 3B and C). Co-treatment with calmodulin (CaM), a specific MLCK activator, showed no significant modulation of FLX-triggered BC deformations (Fig. 3D and E), indicating that MLCK was not implicated in FLX-induced effects. Meanwhile, co-treatment with CaM counteracted BC dilatation induced by ML-9, a specific inhibitor of MLCK (Supplementary Fig. 2). Moreover, incubation of isolated ROCK enzyme with FLX showed no significant effect of the antibiotic on ROCK activity (data not shown), indicating that inhibition of ROCK by FLX required other upstream regulators that were absent in the isolated enzyme system.

### HSP27 is a central mediator of FLX-induced cholestasis

#### Deregulation of HSP27

**Figure 4:**
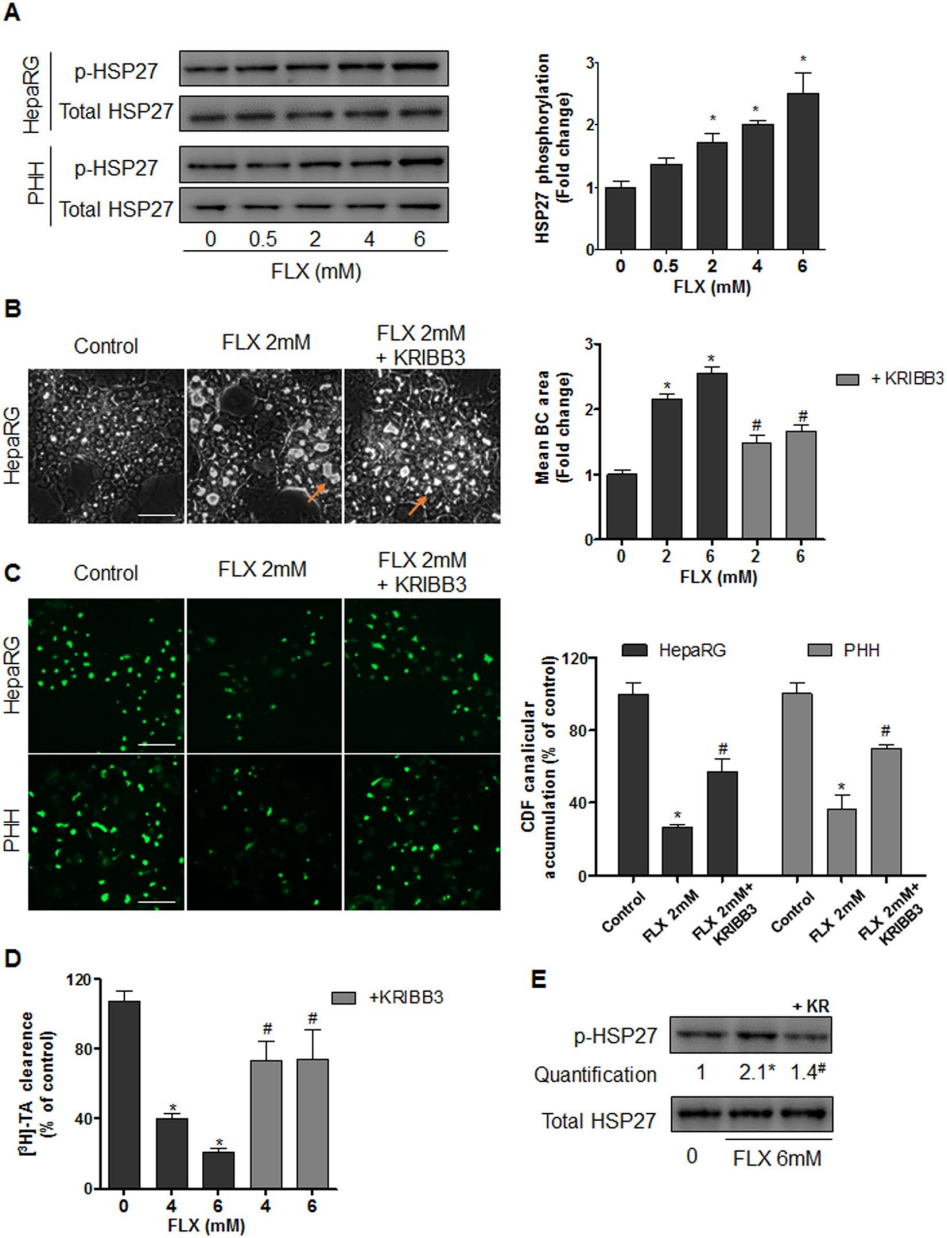
Involvement of HSP27 in FLX-induced effects. (**A**) Representative western blots of the p-HSP27/total HSP27 forms after 2h-treatment with FLX (0–6 mM) in HepaRG cells and PHH. Quantification of HSP27 phosphorylation in HepaRG cells using ImageJ 1.48 software. The displayed blots were cropped and the original full-length gels are included in the supplementary information. (**B**) Representative phase-contrast images of HepaRG cells treated with 2 mM FLX alone or combined with 0.5 µM KRIBB3 after 2 h. Quantification of BC area using ImageJ 1.48 software. Orange arrows indicate BC (bar = 50 μm). (**C**) CDF efflux in HepaRG hepatocytes and PHH treated with 2 mM FLX alone or combined with 0.5 µM KRIBB3 compared to untreated cells. Quantification of CDF accumulation in BC of HepaRG hepatocytes and PHH after 2 h treatment with 2 mM FLX ± 0.5 µM KRIBB3 using ImageJ 1.48 software. (**D**) [^3^H]-TA clearance in HepaRG cells treated with 4 or 6 mM FLX alone or co-treated with 0.5 µM KRIBB3 for 2 h. (**E**) Representative western blots of p-HSP27/total HSP27 forms after 2 h-treatment with 6 mM FLX ± 0.5 µM KRIBB3 (KR). Data were expressed relative to those of untreated cells arbitrarily set at 1 or 100%. They represent the means ± SEM of 3 independent experiments. *p < 0.05 compared with that of untreated cells, ^#^p < 0.05 compared with that of cultures treated with FLX alone.

**Figure 5:**
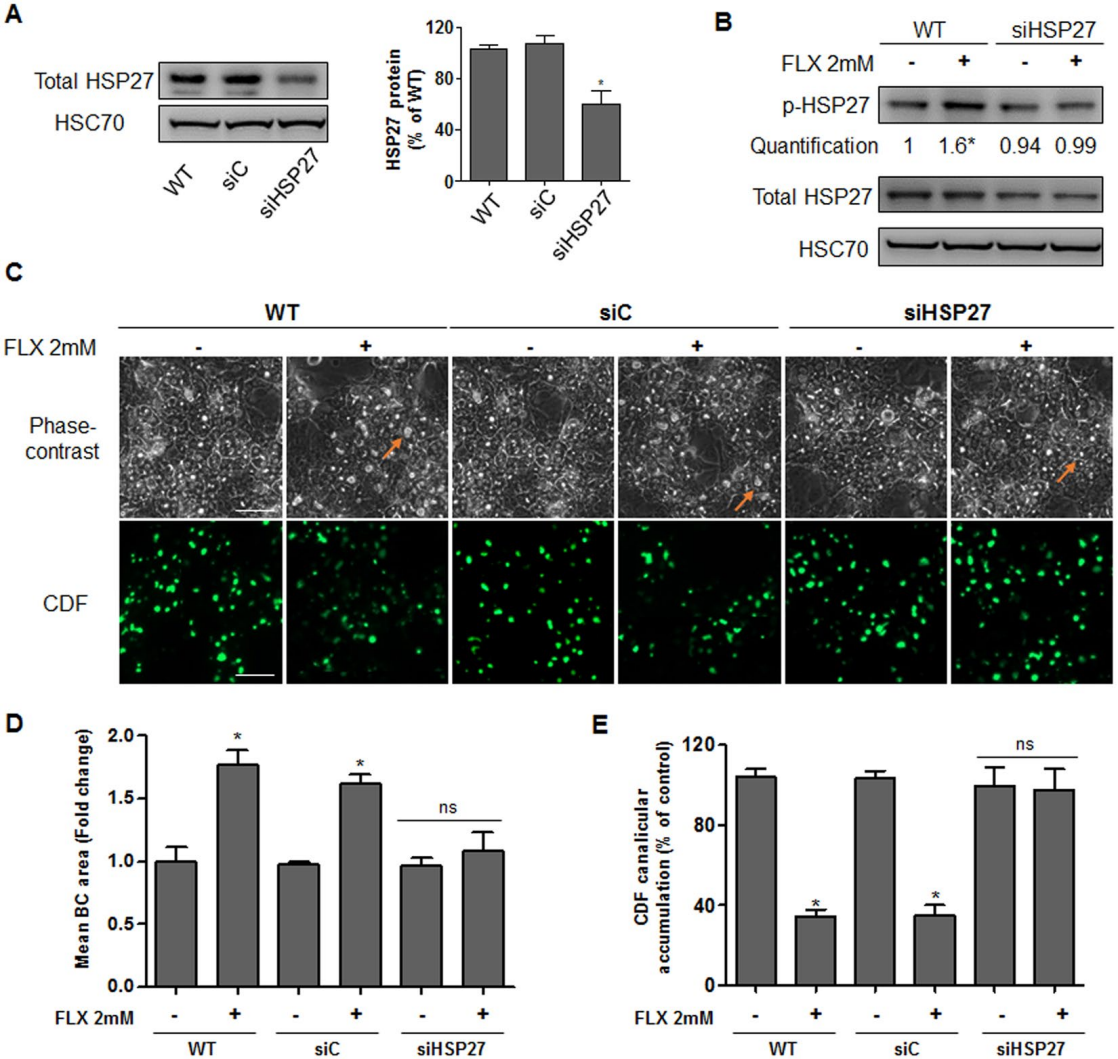
FLX effects in siHSP27-transfected cells. (**A**) Representative western blots of total HSP27 and HSC70 after 72 h in HepaRG cells: wild type (WT), transfected with scramble siRNA (siC) and HSP27 siRNA (siHSP27). Quantification of total HSP27 using ImageJ 1.48 software. (**B**) Representative western blots of the p-HSP27/total HSP27 forms and HSC70 after 2h-treatment with 2 mM FLX in WT and siHSP27 transfected HepaRG cells. The displayed blots were cropped and the original full-length gels are included in the supplementary information. (**C**) Representative phase-contrast images and CDF efflux in wild type, siC and siHSP27 transfected HepaRG cells treated with 2 mM FLX (bar = 50 μm). (**D**) Quantification of the BC area and (**E**) canalicular CDF accumulation in HepaRG cells after 2 h of FLX treatment using ImageJ 1.48 software. Data were expressed relative to those of the untreated cells arbitrarily set at a value of 1 or 100%. They represent the means ± SEM of 3 independent experiments. *p < 0.05 compared with that of untreated cells.

We speculated that effects of FLX on ROCK were indirect and involved other cellular molecular pathways. Among them, HSP27 plays different roles in the regulation of actin cytoskeleton dynamics and can form a complex with ROCK^28^. Western blot analysis showed a dose-dependent increase in HSP27 phosphorylation after 2 h FLX treatment in both HepaRG cells and PHH. Total HSP27 content remained unchanged (Fig. 4A). Addition of 0.5 µM KRIBB3, an inhibitor of HSP27 phosphorylation, prevented the increase of FLX-triggered p-HSP27 and reduced FLX-triggered BC dilatation by 58% compared to FLX alone. Furthermore, co-treatment with KRIBB3, restored by 50–70% the decrease in the efflux of both CDF and [^3^H]-TA (Fig. 4B–E). To confirm the role of HSP27 in these effects, specific siRNA targeting HSP27 mRNA was used and found to inhibit by 60% total HSP27 protein expression (Fig. 5A). After transfection with siHSP27, HepaRG cells were exposed or not to FLX. In siHSP27-transfected cells, no significant induction of HSP27 phosphorylation was observed and FLX failed to induce BC dilatation and inhibition of CDF efflux (Fig. 5B–E).

#### HSP27 phosphorylation involves PKC/P38

**Figure 6:**
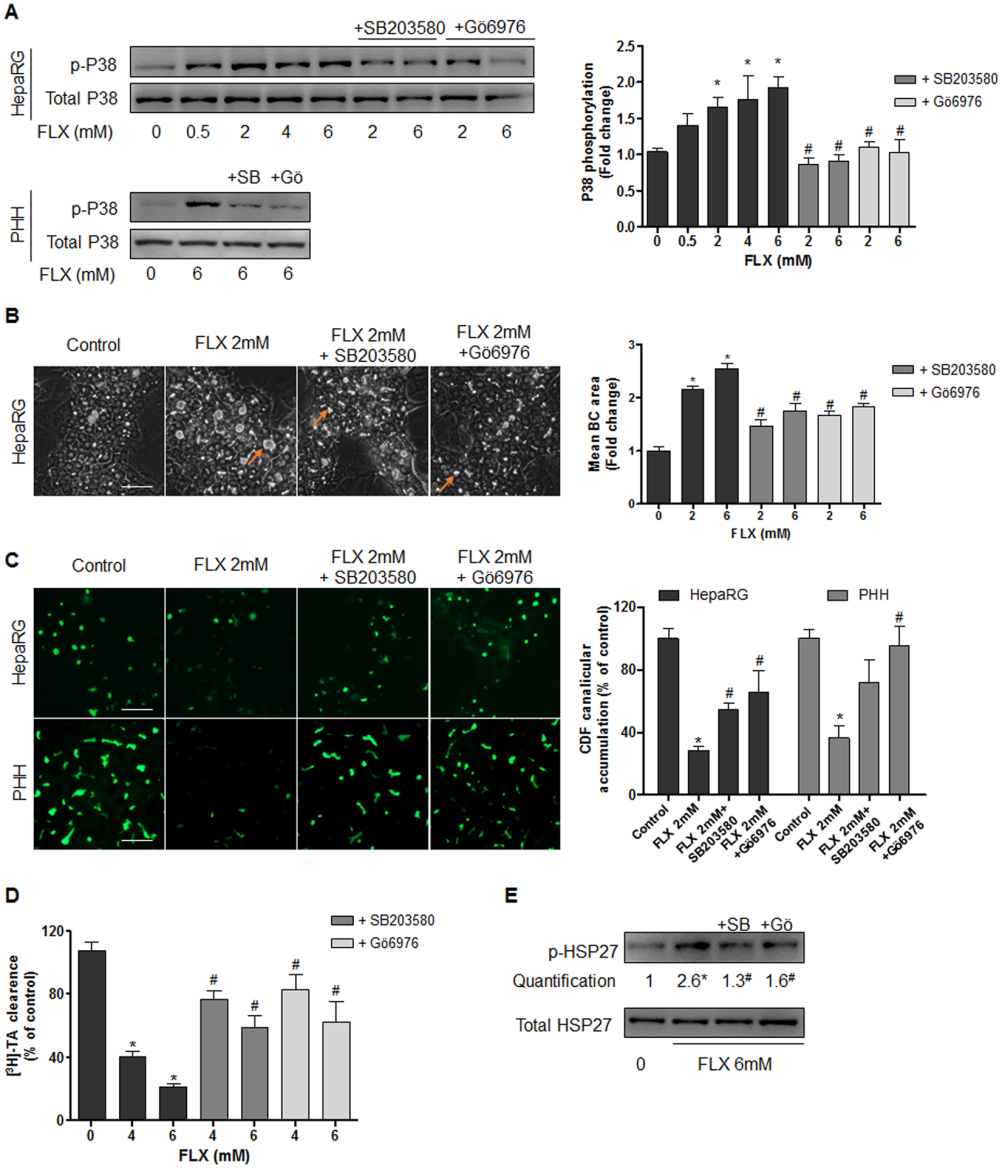
Involvement of PKC/P38 pathway in FLX-induced effects. (**A**) Representative western blots of the p-P38/total P38 forms after 2h-treatment with FLX (0–6 mM) alone or combined with 20 µM PKC inhibitor (Gö6976; Gö) or 10 µM P38 inhibitor (SB203580; SB) in HepaRG cells and PHH. Quantification of p-P38 in HepaRG cells using ImageJ 1.48 software. The displayed blots were cropped and the original full-length gels are included in the supplementary information. (**B**) Representative phase-contrast images of HepaRG cells treated with 2 mM FLX alone or combined with 20 µM Gö6976 or 10 µM SB203580. Quantification of BC area using ImageJ 1.48 software. Orange arrows indicating BC deformation (bar = 50 μm). (**C**) CDF efflux in HepaRG hepatocytes and PHH treated 2 h with 2 mM FLX alone or combined with 20 µM Gö6976 or 10 µM SB203580. Quantification of CDF accumulation in BC of HepaRG hepatocytes and PHH, using ImageJ 1.48 software. (**D**) [^3^H]-TA clearance in HepaRG cells treated with 4 or 6 mM FLX alone or co-treated with 20 µM Gö6976 or 10 µM SB203580 for 2 h. (**E**) Representative western blots of p-HSP27/total HSP27 forms after 2h-treatment with 6 mM FLX alone or combined with 10 µM P38 inhibitor (SB203580; SB) or 20 µM PKC inhibitor (Gö6976; Gö). Data were expressed relative to those of untreated cells arbitrarily set at 1 or 100%. They represent the means ± SEM of 3 independent experiments. *p < 0.05 compared with that of untreated cells, ^#^p < 0.05 compared with that of cultures treated with FLX alone.

Then, we focused on the identification of the sequential events leading to HSP27 activation. It is recognized that HSP27 is a downstream substrate of the p38 MAPK cascade^29, 30^. Therefore, we analyzed whether PKC and its downstream effector P38 were involved in FLX-induced HSP27-dependent cholestatic effects. Western blots showed a dose-dependent increase in P38 phosphorylation after 2 h FLX treatment. This increase was prevented by co-treatment with the P38 inhibitor SB203580 in both HepaRG cells and PHH. Similarly, the PKCα and β inhibitor, Gö6976, prevented the increase in p-P38, indicating that activation of P38 by FLX depended on PKC (Fig. 6A). Both inhibitors reduced BC dilatation (Fig. 6B) and restored partially the efflux of CDF and [^3^H]-TA (Fig. 6C and D). To investigate whether PKC/P38 activation led to FLX-induced activation of the HSP27 signaling pathway, both SB203580 and Gö6976 were used and their effects on FLX-induced HSP27 activation were examined. Co-treatment with SB203580 or Gö6976 remarkably abolished FLX-induced HSP27 activation (Fig. 6E). These results indicated that FLX-induced HSP27 was dependent on activation of the PKC/P38 pathway.

### The HSP-dependent stress signal leads to PI3K/AKT activation

#### FLX induces PI3K/AKT activation

**Figure 7:**
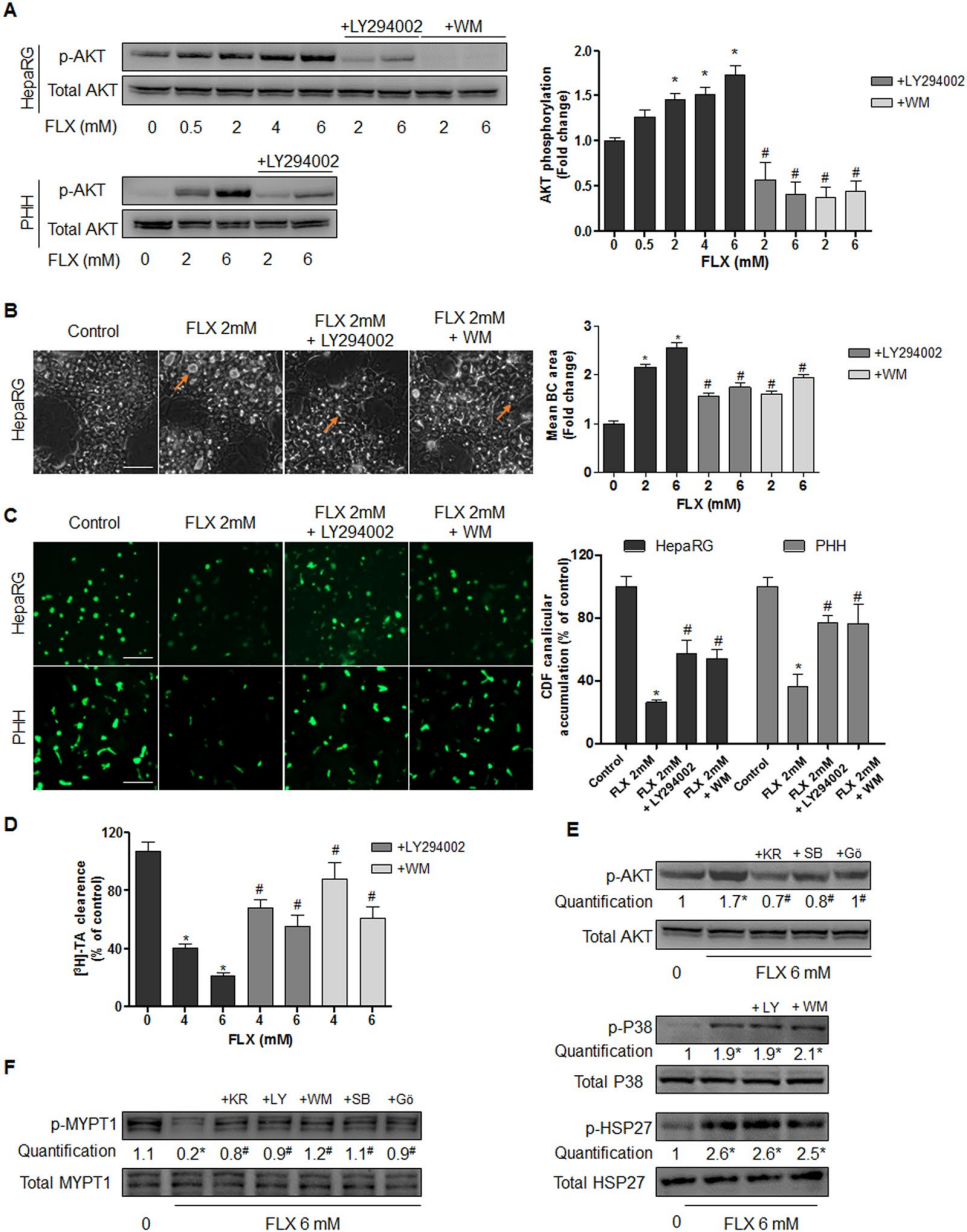
Involvement of PI3K/AKT pathway in FLX-induced effects. (**A**) Representative western blots of p-AKT/total AKT forms after 2h-treatment with FLX (0–6 mM) alone or combined with the PI3K inhibitors LY294002 (10 µM) or WM (0.25 µM) in HepaRG cells and PHH. Quantification of p-AKT in HepaRG cells using ImageJ 1.48 software. (**B**) Representative phase-contrast images of HepaRG cells treated for 2 h with 2 mM FLX alone or combined with 10 µM LY294002 or 0.25 µM WM. Quantification of BC area using ImageJ 1.48 software. Orange arrows indicating BC deformation (bar = 50 μm). (**C**) CDF efflux in HepaRG hepatocytes and PHH treated for 2 h with 2 mM FLX alone or combined with 10 µM LY294002 or 0.25 µM WM. Quantification of CDF accumulation in BC of HepaRG hepatocytes and PHH, using ImageJ 1.48 software. (**D**) [^3^H]-TA clearance in HepaRG cells treated with 4 or 6 mM FLX alone or co-treated with 10 µM Y294002 or 0.25 µM WM for 2 h. (**E**) Representative western blots of p-AKT/total AKT forms after 2 h treatment with 6 mM FLX alone or combined with 0.5 µM HSP27 inhibitor (KRIBB3; KR), 10 µM P38 inhibitor (SB203580; SB), and 20 µM PKC inhibitor (Gö6976; Gö). Representative western blots of p-P38/total P38 and p-HSP27/total HSP27 after 2 h treatment with 6 mM FLX alone or combined with the PI3K inhibitors 10 µM LY294002 (LY) or 0.25 µM WM. (**F**) Representative western blots of p-MYPT1/total MYPT1 after 4 h treatment with 6 mM FLX alone or combined with KR, LY, WM, SB or Gö. The displayed blots were cropped and the original full-length gels are included in the supplementary information. Data were expressed relative to those of untreated cells arbitrarily set at 1 or 100%. They represent the means ± SEM of 3 independent experiments. *p < 0.05 compared with that of untreated cells, ^#^p < 0.05 compared with that of cultures treated with FLX alone.

Previous studies have shown that stress-induced HSP27 activates PI3K/AKT^26^. So we hypothesized the implication of the PI3K/AKT pathway. Western blots of AKT, the downstream effector of PI3K, showed that FLX increased p-AKT phosphorylation in a dose-dependent manner in both HepaRG cells and PHH after 2 h (Fig. 6A and B). The total AKT content remained unchanged. Co-treatment with the PI3K inhibitors LY294002 (10 µM) or wortmannin (WM) (0.25 µM) completely prevented p-AKT increase, indicating that activation of AKT depended on PI3K (Fig. 7A). Then, we investigated whether the activated PI3K/AKT pathway was involved in FLX-induced cholestasis. Co-treatment with LY294002 or WM prevented FLX-induced BC dilatation at all tested concentrations in HepaRG cells (Fig. 7B), and restored partially FLX-induced decrease in CDF and [^3^H]-TA efflux (Fig. 7C,D).

#### PI3K/AKT activation is dependent on HSP27

To determine whether PI3K/AKT acted downstream of HSP27 activation by FLX, HSP27 activity was blocked by treating HepaRG cells with KRIBB3 and AKT stimulation with FLX was examined. The results showed that co-treatment with 0.5 μM KRIBB3 largely inhibited FLX-induced AKT phosphorylation compared to control cells. Similarly, PKC and P38 inhibitors prevented AKT phosphorylation by FLX. By contrast, PI3K inhibitors did not modulate HSP27 and P38 activation by FLX. These results confirmed that PI3K/AKT acted downstream of HSP27 and PKC/P38 in FLX-induced cholestasis (Fig. 7E).

#### ROCK acts downstream of HSP27-AKT

A final step was to link P38-HSP27-AKT modulation to ROCK activity reduction by FLX. For this purpose, we examined MYPT1 phosphorylation after treatment with FLX alone or combined with PKC, P38, HSP27, and PI3K inhibitors. FLX alone inhibited MYPT1 phosphorylation compared to untreated cells. Interestingly, co-treatment with the 5 different inhibitors (KRIBB3, SB203580, Gö6976, LY294002 and WM) prevented FLX-induced MYPT1 inhibition, suggesting that ROCK inhibition was downstream in the axis (Fig. 7F).

### Other cholestatic PRAs activate HSP27 leading to BC deformations and bile acids efflux failure

**Figure 8:**
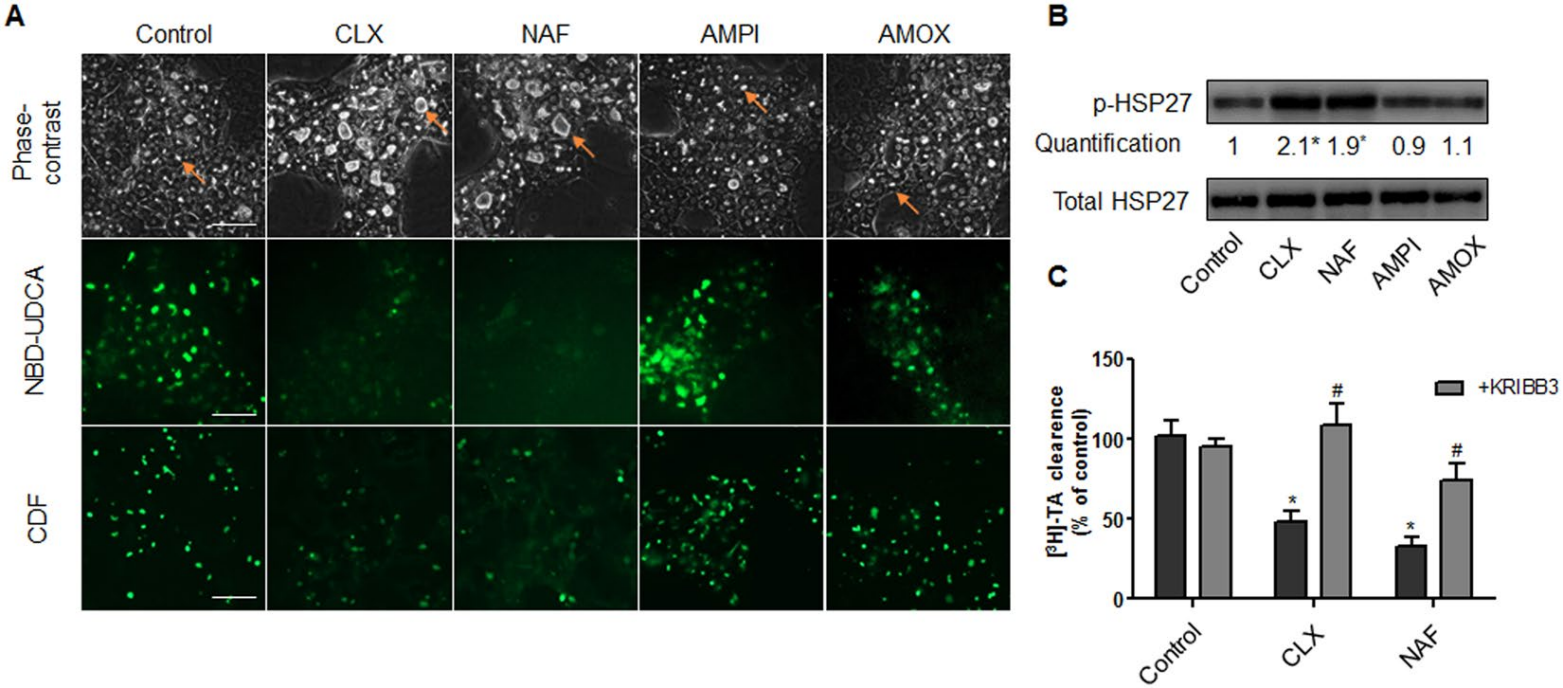
HSP27-dependent cholestatic effects of penicillinase-resistant antibiotics in human HepaRG cells. (**A**) Phase-contrast images, NBD-UDCA and CDF efflux in HepaRG cells either untreated or treated with 6 mM cloxacillin (CLX), nafcillin (NAF), ampicillin (AMPI) and amoxicillin (AMOX) for 2 h. Phase-contrast images were captured using time-lapse microscopy. Orange arrows indicate BC deformations. Fluorescent images were obtained with a Cellomics ArrayScan VTI HCS Reader (bar = 50 µm). (**B**) Representative western blots of the p-HSP27/total HSP27 forms after 2h-treatment of HepaRG cells with 6 mM CLX, NAF, AMPI and AMOX. Quantification of p-HSP27 using ImageJ 1.48 software. The displayed blots were cropped and the original full-length gels are included in the supplementary information. (**C**) [^3^H]-TA clearance in HepaRG cells treated with 6 mM CLX or NAF alone or co-treated with 0.5 µM KRIBB3 for 2 h. Data were expressed relative to those of untreated cells arbitrarily set at a value of 1 or 100%. They represent the means ± SEM of 3 independent experiments. *p < 0.05 compared with that of untreated cells.

Then, we compared the effects of FLX to other members of the β-lactam PRAs that are also known to be cholestatic in clinic, namely cloxacillin and nafcillin. As observed with FLX, these two antibiotics induced BC dilatation within 2 h treatment and inhibited NBD-UDCA and CDF canalicular efflux (Fig. 8A). Western blot analysis also showed a 2-fold induction of HSP27 phosphorylation after 2 h treatment (Fig. 8B). As expected, addition of KRIBB3 protected against BC dilatation and reduced inhibition of transporters (Fig. 8C). The specificity of our data on PRAs, was confirmed by the demonstration that two non cholestatic non-PRAs, ampicillin and amoxicillin, did not alter BC morphology and did not modulate HSP27 phosphorylation (Fig. 8A,B).

## Discussion

The penicillinase-resistant β-lactam antibiotic family, especially FLX, is known to cause severe liver injury. Female sex, age, high daily doses and HLA-B*57:01 allele have been shown to be associated with higher risk of liver injury due to FLX. However, whether this antibiotic is able to induce cholestasis directly in the hepatocytes without an immune influence has not been clearly established yet. In this work, we characterized for the first time the sequential molecular events involved in FLX-induced cholestasis in absence of an immune reaction and provided several shreds of evidence for a crucial role of the HSP27 chaperone protein.

Recently, we showed that bile canaliculi undergo contraction/relaxation movements which are essential for bile flow. Disruption of BC dynamics by certain drugs is associated with occurrence of cholestasis injury^24^. If few cholestatic drugs such as cyclosporine A^31, 32^ and chlorpromazine^33^, induced constriction of BC associated with irreversible severe damage and cell death, others covering a wider range of cholestatic compounds, induced dilatation^24, 34^. Importantly, BC dilatation is observed in patients suffering from cholestasis^35, 36^. In the current work, we showed that FLX also caused BC dilatation associated with disruption of canalicular bile flow as demonstrated by reduction in [^3^H]-TA efflux and fluorescent substrates NBD-UDCA and CDF trafficking to BC lumen. Noteworthy, all these morphological and functional alterations were observed at non-cytotoxic concentrations. Indeed, FLX-induced cholestatic effects appeared at 0.5 mM, while cytotoxic effects were observed at concentrations of 12 mM or higher in HepaRG hepatocytes. However, FLX was more cytotoxic to the primitive biliary-like HepaRG cell population. This higher sensibility could be attributed to the lack of detoxifying enzymes in these cells^32^ or the release of FLX reactive metabolites by HepaRG hepatocytes. In support, FLX OH-metabolite formed by a CYP3A4-mediated reaction was reported to be cytotoxic to biliary epithelial cells after its excretion into the bile duct^37^.

Several studies have shown that human exposure to FLX is associated with rare and delayed hypersensitivity reactions characterized by the recruitment of CD8+ cells to kill the hepatocytes, and favored in individuals carrying HLA-B*57:01 determinant, but at a frequency not exceeding 1/1000^14, 38^. However, there is no evidence for the role of these immune cells in the development of cholestasis. Our data demonstrate that FLX can induce cholestasis independently of an immune reaction and prior to hepatocellular injury. In support, no induction of inflammatory mediators was detected after treatment with FLX. It could suggest that in clinic cholestatic insult is a primary and frequent event which in susceptible patients, could lead to hepatocellular injury through triggering chemokine production and recruitment of immune cells^39, 40^. This paradigm is supported by epidemiological studies showing that cholestasis accounts for 95% of patients with FLX-induced liver injury^41^; meanwhile hepatocellular injury alone is extremely rare.

The dynamic movements of BC involve signaling mechanisms controlled by ROCK or MLCK that regulate acto-myosin interactions. Disrupting this pathway with cholestatic drugs resulted in BC deformations and bile flow secretory failure^24^. Several evidences showed the implication of ROCK in FLX effects: i) the dose-dependent inhibition of ROCK activity at concentrations of FLX that caused dilatation of BC and ii) the decrease of MYPT1 phosphorylation responsible for permanent relaxation of the actin filaments surrounding BC. Furthermore, the MLCK activator calmodulin was ineffective, indicating that ROCK and not MLCK was mainly implicated in BC deformations caused by FLX. By considering Cmax values measured in patients, i.e., 0.3–0.8 mM after only a single dose of FLX^42^, these data provided clear arguments that FLX can induce cholestasic effects in cultured hepatocytes at therapeutic doses.

HSPs, mainly 27, 70 and 90, are known to play a crucial cytoprotective role in conditions such as oxidative and endoplasmic reticulum stress, immune reactions and abnormal protein folding. Targeting HSP27 is thought to represent a novel promising strategy for the treatment of patients with liver injury^43^. Due to its structural organization, HSP27 can act as a sensor and allows cells to adapt and eventually overcome lethal conditions, by interacting with appropriate protein partners such as actin, procaspase-3 and AKT^44^. Therefore, undoubtedly, HSP27 can allow the cells to rapidly overcome a stress and restore homeostasis. However, whether these protective beneficial effects could be accompanied by impairment of certain physiological processes leading to DILI remained to be clarified. Aberrations in HSP27 protein phosphorylation have been closely linked to major diseases such as renal injury and fibrosis, cancer, neuro-degenerative and cardiovascular diseases^45^. However, no study had shown the association of this chaperone with cholestatic liver injury. Evidence was provided that HSP27 forms a complex with ROCK to control cytoskeletal contraction/relaxation dynamics^21, 22, 28^, leading us to focus our study on this protein. Several arguments support the crucial role of HSP27 in FLX-induced adverse cholestatic effects through regulating ROCK activity: (i) FLX induced HSP27 phosphorylation in a dose-dependent manner; (ii) the HSP27 inhibitor KRIBB3 protected against FLX-induced BC dilatation; (iii) KRIBB3 restored the reduction of [^3^H]-TA and CDF clearance caused by FLX; (iv) this HSP27 inhibitor also nullified the inhibitory effect of FLX on ROCK; and finally, (v) FLX failed to induce cholestatic effects in siHSP27-transfected HepaRG cells.

Consequently, the potential activators of HSP27 were also examined. Several kinases are known to catalyze phosphorylation of HSP27 such as: MAPKAPK-2 and MAPKAPK-3, PKC, protein kinase D, and cGMP-dependent protein kinase^46–48^. It has been demonstrated that HSP27 can form a multicomponent complex with PKC and P38 and this complex has been shown to be involved in controlling HSP27 phosphorylation and stress-induced apoptosis^25, 27^. Whether the PKC/P38 pathway could be activated by FLX was unknown. Our results clearly revealed that FLX caused an increase in P38 phosphorylation. The selective PKC inhibitor Gö6976 effectively blocked FLX-stimulated P38 phosphorylation indicating that P38 is downstream of PKC. It is important to note that both PKC and P38 inhibitors blocked FLX-triggered HSP27 activation and protected against FLX-induced cholestatic effects. Consequently, we conclude that PKC/P38 is an upstream component that regulates HSP27 and ROCK signaling pathway in FLX-triggered cholestasis. Noticeably, it has been reported that activation of PKC and its downstream modulator P38 pathway are implied in cholestasis induced by estradiol and cyclosporine A, without further investigation of their association with HSP27^31, 49^. All these results lead to postulate for an adverse role of HSP27 in the cholestatic insult.

**Figure 9:**
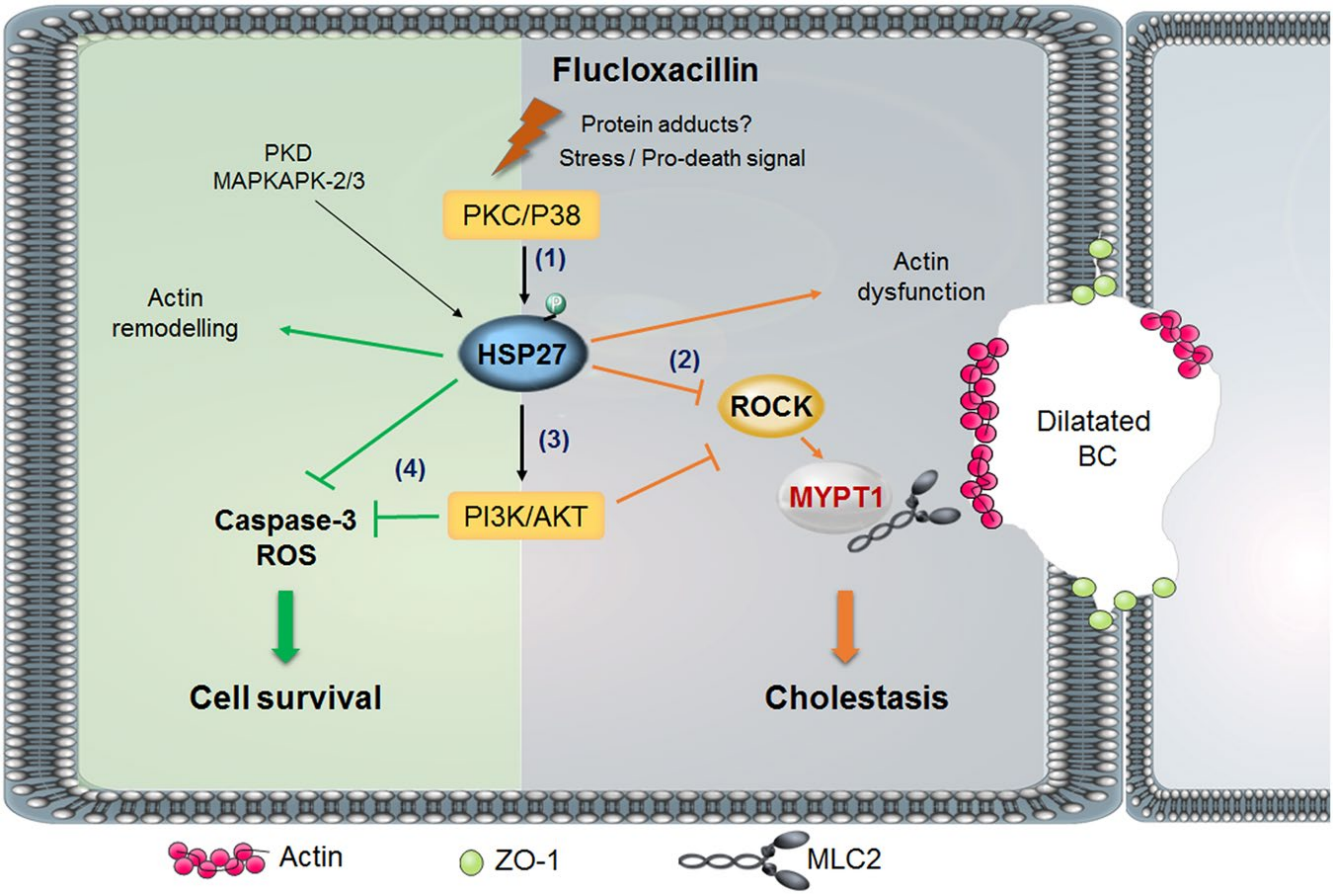
Schematic representation of sequential molecular mechanisms involved in FLX-induced cholestasis. FLX induces phosphorylation of HSP27 through activation of PKC/P38 (1). Activated p-HSP27 plays a central role in cholestasis; it inhibits ROCK and consequently MYPT1 phosphorylation leading to BC dilatation and cholestasis (2) and activates PI3K/AKT leading to BC dilation and cholestasis through MYPT1 dephosphorylation (3). In parallel to cholestatic insult, activated p-HSP27 could lead to cell resistance and survival through PI3K/AKT pathway activation preventing caspases induction and ROS generation (4). ROCK, Rho-kinase; MLC2, myosin light chain 2; MYPT1, myosin phosphatase target subunit 1; BC, bile canaliculi; ZO-1, zona occludens-1 protein; PKC, protein kinase C; P38, p38 mitogen-activated protein kinase; PI3K, phosphoinositide 3-kinase; AKT, protein kinase B; HSP27, heat shock protein 27; PKD, protein kinase D; ROS, reactive oxygen species; MAPKAPK, mitogen-activated protein kinase-activated protein kinase.

Previous studies have shown that stress-induced HSP27 phosphorylation resulted in PI3K activation and AKT phosphorylation^19^. Therefore, it is reasonable to postulate that PI3K/AKT could mediate HSP27-related effects. Our results showed that FLX induced a dose-dependent increase in PI3K/AKT activity as demonstrated by the increase in AKT phosphorylation. Blocking HSP27 by KRIBB3 abrogated FLX-induced AKT activation evidencing that PI3K/AKT activation by FLX is HSP27-dependent. We evidenced a clear contribution of PI3K/AKT in FLX-induced adverse effects. Blocking PI3K activity by LY294002 or WT significantly prevented FLX-caused cholestatic features. These results indicated a HSP27-dependent role of PI3K/AKT in FLX-induced cholestasis. In agreement, involvement of the PI3K/AKT pathway in cholestatic injury has been reported^49^. Even though cholestastic features were observed, the lack of cytotoxicity and enhanced caspase-3 activity at the tested concentrations of FLX could indicate the occurrence of an adaptive protective response that could be mediated by the activated PI3K/AKT. In agreement several studies have shown that HSP27 activates an adaptive mechanism to preserve cell survival through PI3K/AKT^26^. In total, HSP27 could interact with the PI3K/AKT pathway to protect the cells against death, but these interactions could also be responsible for the adverse effects in bile acids secretion (Fig. 9).

Cholestatic features and involvement of the HSP27 signaling pathway observed with FLX were similarly evidenced with other PRAs. Indeed, two other penicillinase-resistant β-lactam antibiotics, cloxacillin and nafcillin, which are known to be cholestatic in patients^2, 7, 50^, also caused BC dilatation and HSP27 activation in HepaRG cells. By contrast, two non-penicillinase-resistant β-lactams antibiotics, ampicillin and amoxicillin, which are known to have extremely low hepatotoxic potential in clinic^7^, did not induce cholestatic effects and did not modulate HSP27 activity in HepaRG cells. Noteworthy, if amoxicillin causes little hepatotoxicity when administered alone; its combination with clavulanic acid markedly increases its risk of hepatotoxicity^51, 52^. Based on the available literature, it appears that PRAs could form albumin-conjugated adducts^53^. However, whether formation of protein adducts is an initial event for these HSP-dependent effects needs further investigations.

In summary, the current study represents the first demonstration that FLX and other PRAs can induce cholestatic effects in human hepatocytes in the absence of an immune reaction. It shows that HSP27, despite its protective function, can have adverse effects as a central mediator in the sequential events leading to ROCK disruption and cholestasis, reinforcing the role of ROCK and highlights HSP27 as a novel mechanism in drug-induced cholestasis. Together, our data support the conclusion that PRAs can cause a non-immune-mediated cholestasis that is not restricted to patients possessing certain genetic determinants.

## Materials and Methods

### Reagents

Flucloxacillin, cloxacillin, ampicillin, amoxicillin, methylthiazoletetrazolium (MTT), 1-(5-Chloronaphthalene-1-sulfonyl)-1H-hexahydro-1,4-diazepine hydrochloride (ML-9), ﻿N-Acetyl-Asp-Glu-Val-Asp-7-amido-4-methylcoumarin﻿ (Ac-DEVD-AMC) and 5(6)-carboxy-2, 7-dichlorofluorescein diacetate (CDFDA) were purchased from Sigma (St. Quentin Fallavier, France). Rho-associated kinase (ROCK) activity assay kit was from Cell Biolabs (San Diego, CA). Human C-reactive protein (CRP), interleukin-6 (IL-6), interleukin-1β (IL-1β) and CXCL8/IL-8 DuoSet kits were from R&D (Abingdon, United Kingdom). 3α-Hydroxy-7 nitrobenzoxadiazolyl-ursodeoxycholic acid (NBD-UDCA) fluorescent analogue was synthesized by ICOA-University of Orleans (Orléans, France). Dichlorodihydrofluorescein (H2-DCFDA) was obtained from Invitrogen Molecular Probe (Saint Aubin, France). Gö6976, LY294002, Wortmannin, SB203580, and bovine brain calmodulin were from Calbiochem (San Diego, CA). Phalloidin fluoprobe was purchased from Interchim (Montluçon, France). [^3^H]-Taurocholic acid ([^3^H]-TA) was from Perkin Elmer (Boston, MA). Specific antibodies against p-AKT, AKT, p-P38, P38, p-HSP27, p-MYPT1 and MYPT1 were purchased from Cell Signalling Technology (Schuttersveld, Netherlands). Nafcillin, KRIBB3, and specific antibodies against HSP27 and HSC70 were purchased from Santa Cruz Biotechnology (Dallas, TX). Anti-zona occludens protein 1 (ZO-1) antibody was obtained from BD Biosciences (Le Pont de Claix, France). Specific antibody against occludin and secondary antibodies were purchased from Invitrogen (Saint Aubin, France). Hoechst dye was from Promega (Madison, Wisconsin). Other chemicals were of reagent grade.

### Cell cultures

HepaRG cells were seeded at a density of 2.6 × 10^4^ cells/cm^2^ in Williams’ E medium supplemented with 2 mM glutamax, 100 U/mL penicillin, 100 µg/mL streptomycin, 10% HyClone fetal calf serum, 5 µg/mL insulin, and 50 µM hydrocortisone hemisuccinate. At confluence (after 2 weeks), HepaRG cells were shifted to the same medium supplemented with 1.7% dimethyl sulfoxide (DMSO) for 2 additional weeks to obtain confluent differentiated cultures containing equal proportions of hepatocyte-like and progenitor/primitive biliary-like cells^54^. These differentiated HepaRG cell cultures were used for the analytical assays.

Human hepatocytes were obtained from Biopredic International (St Gregoire, France), who holds all permits required for the acquisition and transformation of human biological materials to be used in research (AC-2007-43/ law CSP L1245-2/IE-2011-566). Informed consents were obtained from all subjects (Center of Biological Resources, Rennes, France, BB-0033-00,056). Research protocols were performed in accordance with French legal guidelines (French Ministery of Health). All the experimental protocols with human hepatocytes were approved by the local institutional committee (Pontchaillou Hospital, Rennes, France). The hepatocytes were isolated by collagenase perfusion of histologically normal liver fragments from 4 adult donors undergoing resection for primary and secondary tumours. The primary cultures were obtained by hepatocyte seeding at a density of 1.5 × 10^5^ cells/cm^2^ onto collagen-precoated plates in Williams’ E medium supplemented as detailed above without DMSO. The medium was discarded 12 h after cell seeding, and the cultures were then maintained in Williams’ E medium deprived of DMSO and HyClone fetal calf serum.

### Cell treatment

Both cell models were treated with the antibiotics for different time points either alone or combined with the different inhibitors. The cells were pre-treated for 1 h with the inhibitors alone then followed by a co-treatment with FLX ± inhibitors in Williams’ E medium deprived of antibiotics and HyClone fetal calf serum with a final concentration of 0.2% DMSO.

### Cell viability

Cytotoxicity was evaluated by the MTT colorimetric assay. Briefly, the cells were seeded in 96-well plates and treated with various concentrations of FLX in triplicate for 24 h. After removing the medium, 100 μl of serum-free medium containing MTT (0.5 mg/ml) was added to each well and incubated for 2 h at 37 °C. The water-insoluble formazan was dissolved in 100 μl DMSO, and absorbance was measured at 550 nm.

### SiRNA transfection

Transfection was performed using the reverse-transfection method following the protocol of the JetPRIME® transfection reagent (Polyplus Transfection, Illkirch, France). For one well of a 24-well plate, 27pmol of siRNA were mixed with 2 µL of the jetPRIME® reagent in 50 µL of jetPRIME® buffer according to the supplier’s instructions. The mix was placed into the well, and afterward, the cells in 400 µL of medium were added. Seventy-two hours later, the medium was replaced and the cells were exposed or not to FLX. The sequences of small interfering RNA (siRNA) for HSP27 was 5′-UGAGAGACUGCCGCCAAGUAA-3′, the sequence of control siRNA was 5′-UUCUCCGAACGUGUCACGUTT-3′ (Eurogentec, Angers, France).

### Real-time quantitative polymerase chain reaction analysis

Total RNA was extracted from 10^6^ HepaRG cells with the SV total RNA isolation system (Promega). RNAs were reverse transcribed into cDNA and real-time quantitative polymerase chain reaction (RT-qPCR) was performed using a SYBR Green mix. Primer sequences are listed in Supplementary Table 2.

### Caspase-3 activity

After treatment, cells were harvested and stored as pellets at −80 °C. After cell lysis, 40 μg of protein were incubated with 80 μM Ac-DEVD-AMC in caspase-3 activity buffer (20 mM PIPES pH 7.2, 100 mM NaCl, 10 mM dithiotreitol, 1 mM EDTA, 0.1% CHAPS and 10% sucrose) at 37 °C for 1 h. Caspase 3-mediated cleavage of Ac-DEVD-AMC peptide was continuously measured by spectrofluorimetry using excitation/emission wavelengths of 380/440 nm.

### Measurement of ROS

ROS generation was determined by the H2-DCFDA assay. Cells were incubated for 2 h at 37 °C with 2 µM H2-DCFDA; then they were washed with cold phosphate buffered saline (PBS) and scraped in phosphate buffer (10 mM, pH 7.4)/methanol (vol/vol) supplemented with Triton X-100 (0.1%). Fluorescence intensity of cell extracts was determined by spectrofluorimetry using excitation/emission wavelengths of 498/520 nm.

### Cytokines and CRP measurements

IL-6, IL-1β, IL-8 and CRP proteins were measured in cell supernatants after 24 h treatment with 0–8 mM FLX treatment using the IL-6, IL-1β, CXCL8/IL-8 and CRP DuoSet kits (R&D systems), according to the manufacturer’s instructions. Briefly, supernatants were collected at the appropriate time points and stored at −80 °C until analysis; 96-well microplates were coated with capture antibody and incubated overnight. Samples and standards were diluted appropriately and added for 2 hours after a saturation step. Secondary antibody was added for 2 hours after washing. Streptavidin-horseradish peroxidase and its substrate were used for revelation. Optical density was read at 450 nm with wavelength correction. All steps were performed at room temperature.

### Time-lapse cell imaging

Phase-contrast images of the HepaRG cells and PHH were captured by time-lapse phase-contrast videomicroscopy. An inverted microscope (Zeiss Axiovert 200 M), equipped with a thermostatic chamber (37 °C and 5% CO_2_), was used to maintain the cells under normal culture conditions. The images were captured with an AxioCam MRm camera.

### BC area quantification

BC area quantification was based on phase-contrast images. After capturing of images, mean BC areas were determined from 9 zones per condition and in 3 independent experiments using ImageJ 1.48 software after different times of exposure. Briefly, after capturing the time-lapse images, bright objects corresponding to BC were segmented by adjusting the shape and area parameters to exclude non-corresponding objects. The data are presented as the fold change of the BC area in treated cells relative to their corresponding control.

### CDF clearance

After treatment the cells were washed with warm Williams’ E medium without phenol red and incubated in 3 μM CDFDA for 30 min at 37 °C in the same medium used for passive intracellular accumulation. Upon hydrolysis by the intracellular esterases, CDFDA was converted to fluorescent CDF (excitation/emission: 488/509 nm) and directed towards the biliary pole by membrane transporters, particularly MRP2. After washing, imaging was performed using a Cellomics ArrayScan VTI HCS Reader (Thermo Scientific). The number of CDF accumulating BC was quantified using ImageJ 1.48 software.

### NBD-UDCA clearance

The cells were washed with warm Williams’ E medium without phenol red and incubated in 5 μM NBD-UDCA (excitation/emission: 488/509 nm) for 30 min at 37 °C in Williams’ E medium without phenol red. NBD-UDCA is secreted into BC by membrane transporters, mainly BSEP^55^. After washing, the cells were treated for 2 h and imaging was performed using an inverted microscope (Zeiss Axiovert 200 M and AxioCam MRm).

### Taurocholate acid clearance

Cells were first exposed to 43.3 nM [^3^H]-TA for 30 min to induce its intracellular accumulation, washed with standard buffer and then incubated with FLX alone or combined with the different inhibitors for 2 h in a standard buffer containing Ca^2+^ and Mg^2+^. After incubation, the cells were washed and scraped in 0.1 N NaOH, and the remaining radiolabelled substrate was measured through scintillation. [^3^H]-TA clearance was determined based on its accumulation in the cell layers (cells + BC) and calculated relative to the control using the following formula: [^3^H]-TA clearance = [^3^H]-TA accumulation in cell layers_*Control*_/[^3^H]-TA accumulation in cell layer_*Tested compound*_ *100.

### ROCK activity

ROCK activity was measured with a Rho-associated kinase activity assay Kit according to the manufacturer’s protocol with certain modifications. Briefly, HepaRG cells were treated with FLX alone or combined with the different inhibitors. After 4 h, the cells were lysed with a lysis buffer supplemented with anti-protease. The samples were kept overnight at 4 °C and then 90 µL of the lysate was deposited in 96-well multi-strip plates pre-coated with MYPT1 supplied with 10 mM DTT, 2 mM MgCl_2_ and 10 mM ATP for 60 min at 30 °C. An anti-phospho-MYPT1 (Thr696) antibody was added for 1 h, and then goat anti-rabbit IgG HRP secondary antibody was added for another 1 h, and chromogenic substrate tetra-methylbenzidine (TMB) was added for 15 min. Absorbance at 450 nm reflected the relative amount of ROCK activity in the sample, which was evaluated relative to the total protein content of each sample.

### Western blotting analysis

HepaRG cells were treated with FLX alone or co-treated with the different inhibitors. After washing with cold PBS, they were suspended in cell lysis buffer supplemented with protease and phosphatase inhibitors (Roche, Mannheim, Germany). Aliquots containing an equivalent total protein content as determined by the Bradford procedure with bovine serum albumin as the standard were subjected to sodium dodecyl sulfate/12% polyacrylamide gel electrophoresis, electrotransferred to Immobilon-P membranes, and incubated overnight with primary antibodies directed against p-MYPT1 (dilution: 1/500), p-AKT (dilution: 1/2000), total AKT (dilution: 1/2000), p-P38 (dilution: 1/1000), total P38 (dilution: 1/1000), p-HSP27 (dilution: 1/500), total HSP27 (dilution: 1/1000) or HSC70 (dilution: 1/500). After exposure to a horseradish peroxidase conjugated anti-mouse/rabbit antibody (dilution: 1/5000) (Thermo Fisher Scientific, Waltham, MA), the membranes were incubated with a chemiluminescence reagent (Millipore, Billerica, MA) and the bands were then visualized with Fusion-Capt software (Vilber Lourmat, Collégien, France) and quantified using ImageJ 1.48 software.

### Immunolabelling

The cells were washed with warm phosphate buffered saline (PBS), fixed with 4% paraformaldehyde for 20 min, and then washed three times with cold PBS. After paraformaldehyde fixation, the cells were permeabilized for 20 min with 0.3% Triton X-100 in PBS followed by a 1h-incubation in PBS containing 1% bovine serum albumin and 5% normal donkey serum. The cells were then incubated overnight with primary antibody directed against ZO-1 or occludin diluted in PBS containing 1% bovine serum albumin and 5% normal donkey serum. After washing with cold PBS, the cells were incubated for 2 h with rabbit Cy^®^5-labelled secondary antibodies (excitation/emission: 649/670 nm). Finally, the cells were again washed with cold PBS and incubated with a rhodamine-phalloidin fluoroprobe SR101 (200 U/ml) (excitation/emission: 540/565 nm) diluted at 1/100 for F-actin labelling for 20 min. The nuclei were labelled with 5ng/ml Hoechst dye (excitation/emission: 346/460 nm). Immunofluorescence images were captured using a Cellomics ArrayScan VTI HCS Reader (Thermo Scientific, New Hampshire, USA).

### Statistical analysis

A one-way ANOVA with a multiple comparison test (GraphPad Prism 5.00) was performed to compare the data. Each value corresponded to the mean ± SEM of three independent experiments. Data were considered significantly different at p < 0.05.

## Electronic supplementary material

Supplementary document

## References

[1] Lee WM (2003). Drug-induced hepatotoxicity. The New England journal of medicine.

[2] Chalasani, N. *et al*. Causes, clinical features, and outcomes from a prospective study of drug-induced liver injury in the United States. *Gastroenterology* **135**, 1924–1934, 1934 e1921–1924 (2008). 10.1053/j.gastro.2008.09.011 PMC3654244 18955056

[3] Andrade RJ (2008). Idiosyncratic drug hepatotoxicity: a 2008 update. Expert Rev Clin Pharmacol.

[4] Andrade RJ (2005). Drug-induced liver injury: an analysis of 461 incidences submitted to the Spanish registry over a 10-year period. Gastroenterology.

[5] George DK, Crawford DH (1996). Antibacterial-induced hepatotoxicity. Incidence, prevention and management. Drug Saf.

[6] Bjornsson E, Olsson R (2006). Suspected drug-induced liver fatalities reported to the WHO database. Dig Liver Dis.

[7] Andrade RJ, Tulkens PM (2011). Hepatic safety of antibiotics used in primary care. The Journal of antimicrobial chemotherapy.

[8] Polson, J. E. Hepatotoxicity due to antibiotics. *Clin Liver Dis* **11**, 549–561, vi (2007). 10.1016/j.cld.2007.06.009 17723919

[9] Padda MS, Sanchez M, Akhtar AJ, Boyer JL (2011). Drug-induced cholestasis. Hepatology.

[10] Russmann S, Kaye JA, Jick SS, Jick H (2005). Risk of cholestatic liver disease associated with flucloxacillin and flucloxacillin prescribing habits in the UK: cohort study using data from the UK General Practice Research Database. Br J Clin Pharmacol.

[11] Bjornsson E, Olsson R (2005). Outcome and prognostic markers in severe drug-induced liver disease. Hepatology.

[12] Hussaini SH, O’Brien CS, Despott EJ, Dalton HR (2007). Antibiotic therapy: a major cause of drug-induced jaundice in southwest England. Eur J Gastroenterol Hepatol.

[13] Andrews E, Daly AK (2008). Flucloxacillin-induced liver injury. Toxicology.

[14] Daly AK (2009). HLA-B*5701 genotype is a major determinant of drug-induced liver injury due to flucloxacillin. Nature genetics.

[15] Carey MA, van Pelt FN (2005). Immunochemical detection of flucloxacillin adduct formation in livers of treated rats. Toxicology.

[16] Salminen WF, Roberts SM, Fenna M, Voellmy R (1997). Heat shock protein induction in murine liver after acute treatment with cocaine. Hepatology.

[17] Salminen WF, Voellmy R, Roberts SM (1997). Differential heat shock protein induction by acetaminophen and a nonhepatotoxic regioisomer, 3′-hydroxyacetanilide, in mouse liver. J Pharmacol Exp Ther.

[18] Salminen WF, Voellmy R, Roberts SM (1998). Effect of N-acetylcysteine on heat shock protein induction by acetaminophen in mouse liver. J Pharmacol Exp Ther.

[19] Deng W (2016). Heat shock protein 27 downstream of P38-PI3K/Akt signaling antagonizes melatonin-induced apoptosis of SGC-7901 gastric cancer cells. Cancer Cell Int.

[20] Doshi BM, Hightower LE, Lee J (2009). The role of Hsp27 and actin in the regulation of movement in human cancer cells responding to heat shock. Cell Stress Chaperones.

[21] Patil SB, Bitar KN (2006). RhoA- and PKC-alpha-mediated phosphorylation of MYPT and its association with HSP27 in colonic smooth muscle cells. Am J Physiol Gastrointest Liver Physiol.

[22] Patil SB, Pawar MD, Bitar KN (2004). Phosphorylated HSP27 essential for acetylcholine-induced association of RhoA with PKCalpha. Am J Physiol Gastrointest Liver Physiol.

[23] Huot J, Houle F, Spitz DR, Landry J (1996). HSP27 phosphorylation-mediated resistance against actin fragmentation and cell death induced by oxidative stress. Cancer Res.

[24] Sharanek A (2016). Rho-kinase/myosin light chain kinase pathway plays a key role in the impairment of bile canaliculi dynamics induced by cholestatic drugs. Sci Rep.

[25] Zheng C (2006). MAPK-activated protein kinase-2 (MK2)-mediated formation and phosphorylation-regulated dissociation of the signal complex consisting of p38, MK2, Akt, and Hsp27. J Biol Chem.

[26] Wu R (2007). Hsp27 regulates Akt activation and polymorphonuclear leukocyte apoptosis by scaffolding MK2 to Akt signal complex. J Biol Chem.

[27] Rane MJ (2003). Heat shock protein 27 controls apoptosis by regulating Akt activation. J Biol Chem.

[28] El-Yazbi AF, Abd-Elrahman KS, Moreno-Dominguez A (2015). PKC-mediated cerebral vasoconstriction: Role of myosin light chain phosphorylation versus actin cytoskeleton reorganization. Biochem Pharmacol.

[29] Chang L, Karin M (2001). Mammalian MAP kinase signalling cascades. Nature.

[30] Widmann C, Gibson S, Jarpe MB, Johnson GL (1999). Mitogen-activated protein kinase: conservation of a three-kinase module from yeast to human. Physiol Rev.

[31] Sharanek A (2014). Different dose-dependent mechanisms are involved in early cyclosporine a-induced cholestatic effects in hepaRG cells. Toxicol Sci.

[32] Sharanek A (2015). Cellular Accumulation and Toxic Effects of Bile Acids in Cyclosporine A-Treated HepaRG Hepatocytes. Toxicol Sci.

[33] Antherieu S (2013). Oxidative stress plays a major role in chlorpromazine-induced cholestasis in human HepaRG cells. Hepatology.

[34] Burbank MG (2016). Early Alterations of Bile Canaliculi Dynamics and the Rho Kinase/Myosin Light Chain Kinase Pathway Are Characteristics of Drug-Induced Intrahepatic Cholestasis. Drug Metab Dispos.

[35] Chung KW (2002). Increased microfilaments in hepatocytes and biliary ductular cells in cholestatic liver diseases. Journal of Korean medical science.

[36] Imanari H, Kuroda H, Tamura K (1981). Microfilaments around the bile canaliculi in patients with intrahepatic cholestasis. Gastroenterologia Japonica.

[37] Lakehal F (2001). Indirect cytotoxicity of flucloxacillin toward human biliary epithelium via metabolite formation in hepatocytes. Chem Res Toxicol.

[38] Nattrass R (2015). Activation of Flucloxacillin-Specific CD8+ T-Cells With the Potential to Promote Hepatocyte Cytotoxicity in a Mouse Model. Toxicol Sci.

[39] Allen K, Jaeschke H, Copple BL (2011). Bile acids induce inflammatory genes in hepatocytes: a novel mechanism of inflammation during obstructive cholestasis. Am J Pathol.

[40] Yang M (2014). Osteopontin is an initial mediator of inflammation and liver injury during obstructive cholestasis after bile duct ligation in mice. Toxicol Lett.

[41] Devereaux BM, Crawford DH, Purcell P, Powell LW, Roeser HP (1995). Flucloxacillin associated cholestatic hepatitis. An Australian and Swedish epidemic?. Eur J Clin Pharmacol.

[42] Adam D, Koeppe P, Heilmann HD (1983). Pharmacokinetics of amoxicillin and flucloxacillin following the simultaneous intravenous administration of 4 g and 1 g, respectively. Infection.

[43] Bao XQ, Liu GT (2008). Bicyclol: a novel antihepatitis drug with hepatic heat shock protein 27/70-inducing activity and cytoprotective effects in mice. Cell Stress Chaperones.

[44] Arrigo AP, Gibert B (2012). HspB1 dynamic phospho-oligomeric structure dependent interactome as cancer therapeutic target. Curr Mol Med.

[45] Vidyasagar A, Wilson NA, Djamali A (2012). Heat shock protein 27 (HSP27): biomarker of disease and therapeutic target. Fibrogenesis Tissue Repair.

[46] Butt E (2001). Heat shock protein 27 is a substrate of cGMP-dependent protein kinase in intact human platelets: phosphorylation-induced actin polymerization caused by HSP27 mutants. J Biol Chem.

[47] Landry J (1992). Human HSP27 is phosphorylated at serines 78 and 82 by heat shock and mitogen-activated kinases that recognize the same amino acid motif as S6 kinase II. J Biol Chem.

[48] Maizels ET (1998). Heat-shock protein-25/27 phosphorylation by the delta isoform of protein kinase C. Biochem J.

[49] Boaglio AC (2010). Phosphoinositide 3-kinase/protein kinase B signaling pathway is involved in estradiol 17beta-D-glucuronide-induced cholestasis: complementarity with classical protein kinase C. Hepatology.

[50] Olsson R (1992). Liver damage from flucloxacillin, cloxacillin and dicloxacillin. J Hepatol.

[51] Parry MF (1987). The penicillins. Med Clin North Am.

[52] de Abajo FJ, Montero D, Madurga M, Garcia Rodriguez LA (2004). Acute and clinically relevant drug-induced liver injury: a population based case-control study. Br J Clin Pharmacol.

[53] Warbrick EV, Thomas AL, Stejskal V, Coleman JW (1995). An analysis of beta-lactam-derived antigens on spleen cell and serum proteins by ELISA and Western blotting. Allergy.

[54] Gripon P (2002). Infection of a human hepatoma cell line by hepatitis B virus. Proc Natl Acad Sci USA.

[55] Wang R (2013). Defective canalicular transport and toxicity of dietary ursodeoxycholic acid in the abcb11−/− mouse: transport and gene expression studies. Am J Physiol Gastrointest Liver Physiol.

