# Supplementary material for: Penicillinase-resistant antibiotics induce non-immune-mediated cholestasis through HSP27 activation associated with PKC/P38 and PI3K/AKT signaling pathways
Source: Sci Rep. 2017 May 12;7:1815. doi: 10.1038/s41598-017-01171-y (PMC5431934; doi:10.1038/s41598-017-01171-y)
Supplement: Supplementary file 1 — Supplementary document [file 41598_2017_1171_MOESM1_ESM.pdf]

Supplementary information

Penicillinase-resistant antibiotics induce non-immune-mediated cholestasis through HSP27 activation associated with PKC/P38 and PI3K/AKT signaling pathways

Audrey Burban[1,2], Ahmad Sharanek[1,2], Romain Hüe[1,2], Marion Gay[3], Sylvain Routier[3], André Guillouzo[1,2 †] and Christiane Guguen-Guillouzo[1,2 †]

[1] INSERM U991, Liver Metabolisms and Cancer, Rennes, France

[2] Rennes 1 University, Rennes, France

[3] ICOA, University of Orleans, UMR CNRS 7311 Orléans France

E-mail addresses: burban.au@gmail.com (A.Burban), ahmad_sharanek@hotmail.com (A.Sharanek), romain.hüe@univ-rennes1.fr (R. Hüe), marion.gay@univ-orleans.fr (M. Gay), sylvain.routier@univ-orleans.fr (S. Routier)

[†] Senior co-authors: correspondence should be addressed at INSERM UMR 991, Université de Rennes 1, Faculté de Pharmacie, F-35043 Rennes cedex, France. Tel.: (33).2.23.23.53.51; andre.guillouzo@univ-rennes1.fr (A. Guillouzo) and christiane.guillouzo@univ-rennes1.fr (C. Guguen-Guillouzo).

Supplementary Table 1. Effects of FLX on expression of mRNAs encoding genes related to hepatobiliary transporters, ROS, and ER stress markers in HepaRG cells.

	FLX (mM)				
**Gene**	**0.5**	**2**	**4**	**6**	**8**
**ER stress markers (6h)**					
CHOP	0.9±0.06	0.93±0.02	0.9±0.02	<u>1.87±0.04</u>	<u>4.34±0.24</u>
ATF4	1.06±0.07	1.29±0.07	1.30±0.08	<u>1.99±0.26</u>	<u>2.02±0.12</u>
ATF6	0.98±0.04	0.97±0.03	0.93±0.03	1.03±0.1	0.95±0.1
GRP78	0.95±0.02	0.91±0.01	0.78±0.04	0.88±0.11	<u>1.52±0.001</u>
**ROS markers (6h)**					
HO-1	1.25±0.11	1.66±0.15	2.21±0.24	<u>2.60±0.39</u>	<u>3.01±0.69</u>
MnSoD	0.97±0.05	0.96±0.08	0.94±0.06	0.90±0.08	1.03±0.08
**Hepatobiliary Transporters (6h)**					
BSEP	0.98±0.09	1.07±0.09	1.16±0.15	1.12±0.08	1.23±0.15
NTCP	0.85±0.06	0.94±0.09	0.84±0.03	0.84±0.11	0.74±0.08
MRP2	1.22±0.16	1.26±0.10	1.42±0.12	1.45±0.15	1.63±0.17
MRP3	0.89±0.05	0.97±0.02	1.05±0.06	1.00±0.6	1.02±0.05
MRP4	0.96±0.06	0.95±0.02	1.08±0.06	1.13±0.05	1.29±0.02
MDR1	1.15±0.02	<u>1.54±0.07</u>	<u>1.66±0.05</u>	<u>1.92±0.16</u>	<u>2.05±0.12</u>
MDR3	0.87±0.04	0.94±0.06	1.06±0.14	1.10±0.14	1.12±0.12
**Hepatobiliary Transporters (24h)**					
BSEP	<u>0.66±0.05</u>	<u>0.46±0.01</u>	<u>0.39±0.02</u>	<u>0.38±0.03</u>	<u>0.34±0.04</u>
NTCP	0.94±0.08	<u>0.57±0.07</u>	<u>0.39±0.07</u>	<u>0.25±0.07</u>	<u>0.20±0.07</u>
MRP2	<u>1.45±0.05</u>	<u>1.55±0.1</u>	<u>1.61±0.1</u>	<u>1.64±0.14</u>	<u>1.79±0.18</u>

MRP3	1.03±0.05	0.88±0.05	0.84±0.08	0.85±0.09	0.92±0.09
MRP4	1.22±0.05	1.27±0.07	1.34±0.07	1.73±0.18	2.10±0.36
MDR1	2.11±0.12	2.26±0.17	2.02±0.20	1.96±0.21	2.33±0.40
MDR3	0.75±0.1	0.59±0.09	0.49±0.08	0.40±0.06	0.38±0.04

Data represent the means ± SEM of three independent experiments. All results are expressed relative to the levels found in untreated cells, arbitrarily set at a value of 1. Underlined values are significant (*p <0.05) compared with non-treated cells.

Supplementary Table 2: Primers sequences for RT-qPCR

Gene	Name	Forward Primer	Reverse Primer
GAPDH	Glyceraldehyde -3-Phosphate dehydrogenase	TTCACCACCATGGAGAAGGC	GGCATGGACTGTGGTCATGA
ATF4	Activating Transcription Factor 4	TGGCATGGTTTCCAGGTCATCT	CCAACAACAGCAAGGAGGATGC
ATF6	Activating Transcription Factor 6	CTGCACCCACTAAAGGCCAGA	GAGGGCAGAACTCCAGGTGCT
CHOP	C/Ebp-Homologous protein	ATGGCAGCTGAGTCATT	AGAAGCAGGGTCAAGAGTGGT
GRP78	Glucose Regulated Protein,78KD	GTTCTTGCCGTTCAAGGTGG	TGGTACAGTAACAACTGCATG
HO1	heme oxygenase 1	ACTTTCAGAAGGGCCAGGT	TTGTTGCGCTCAATCTCCT
MnSOD	manganese superoxide dismutase	GGGTTGGCTTGGTTTCAATA	CTGATTTGGACAAGCAGCAA
BSEP	bile salt export pump	TGATCCTGATCAAGGGAAGG	TGGTTCCTGGGAAACAATTC
MDR3	multidrug resistance protein 3	GGCTTCAGCCGGCATTTTCA	GCAGCATCTGTGGCAAGTCT
MDR1	multidrug resistance protein 1	GCCAAAGCCAAAATATCAGC	TTCCAATGTGTTCGGCATTA
MRP2	multidrug resistance-associated protein 2	TGAGCAAGTTTGAAACGCACAT	AGCTCTTCTCCTGCCGTCTCT
MRP3	multidrug resistance-associated protein 3	GTCCGCAGAATGGACTTGAT	TCACCACTTGGGGATCATTT
MRP4	multidrug resistance-associated protein 4	GCTCAGGTTGCCTATGTGCT	CGGTTACATTTCCTCCTCCA
NTCP	Na^+-dependent taurocholic co-transporting polypeptide	GGGACATGAACCTCAGCATT	CGTTTGGATTTGAGGACGAT

Supplementary Figure 1

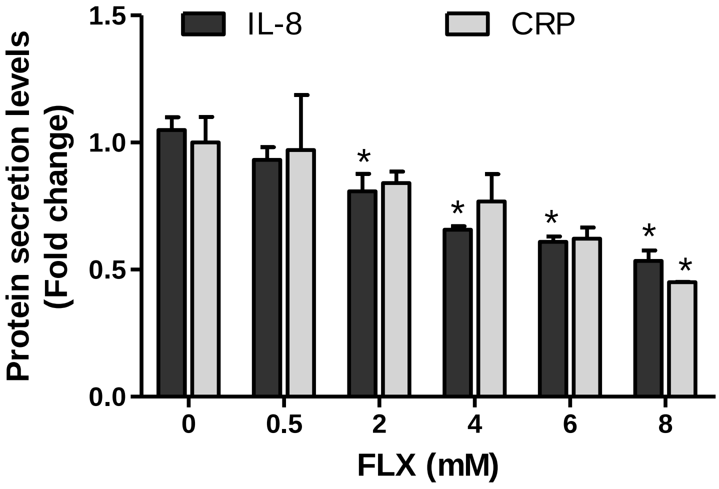

Effects of FLX on inflammatory stress markers. HepaRG cells were treated with 0-8mM FLX for 24h. IL-8 and CRP proteins were measured by ELISA in culture media. Data represent the mean ± SEM of three independent experiments. All results are expressed relative to the levels found in control cells, arbitrarily set at a value of 1. *$P < 0.05$ compared with untreated cells.

Supplementary Figure 2

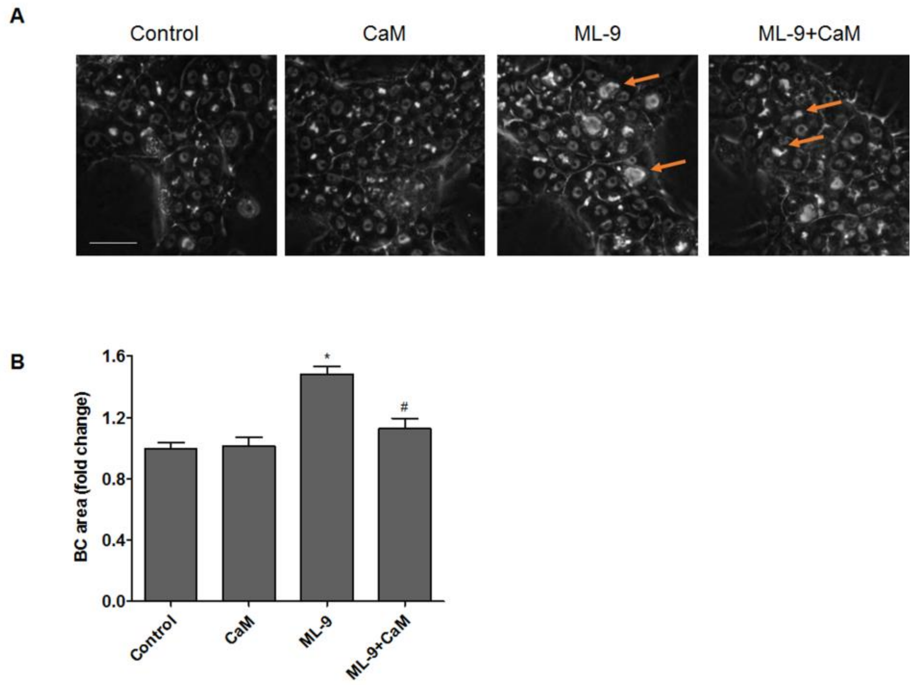

Involvement of MLCK in BC dilatation. (A) Representative phase-contrast images of HepaRG cells treated with 20µM ML-9, a MLCK inhibitor, alone or combined with 5µM CaM, a MLCK activator. Orange arrows indicating BC (bar=50µm). **(B)** Quantification of BC area after 2h using ImageJ 1.48 software. Data were expressed relative to those of the untreated cells arbitrary set at a value of 1. They represent the means ± SEM of 3 independent experiments. *$p < 0.05$ compared with that of controls. $^{\#}p < 0.05$ compared with that of cultures treated with ML-9 alone.
